# Predictors of female sexual dysfunction: a systematic review and qualitative analysis through gender inequality paradigms

**DOI:** 10.1186/s12905-018-0602-4

**Published:** 2018-06-22

**Authors:** Megan McCool-Myers, Melissa Theurich, Andrea Zuelke, Helge Knuettel, Christian Apfelbacher

**Affiliations:** 1 Medical Sociology, Department of Epidemiology and Preventive Medicine, University of Regensburg, Dr. Gessler Strasse 17, 93051 Regensburg, Germany; 2 LMU - Ludwig-Maximilians-Universität Munich, Div Metabolic and Nutritional Medicine, Dr von Hauner Children’s Hospital, Univ. of Munich Medical Center, Lindwurmstr. 4, 80337 Munich, Germany; 3 Institute of Sociology, Friedrich-Schiller-University of Jena, Fürstengraben 1, 07737 Jena, Germany; 4 University Library of Regensburg, Universitaetsstrasse 3, 93053 Regensburg, Germany

**Keywords:** Female sexual dysfunction, Female sexual disorders, Premenopausal, Reproductive-age, Prevalence, Predictors, Risk factors, Gender inequality, Narrative synthesis

## Abstract

**Background:**

Female sexual dysfunction affects 41% of reproductive-age women worldwide, making it a highly prevalent medical issue. Predictors of female sexual dysfunction are multifaceted and vary from country to country. A synthesis of potential risk factors and protective factors may aid healthcare practitioners in identifying populations at risk, in addition to revealing modifiable factors to prevent sexual dysfunction among reproductive-age women.

**Methods:**

Observational studies which assessed the prevalence and predictors of female sexual dysfunction in reproductive-age women were systematically sought in relevant databases (2000–2014). Significant predictors were extracted from each included publication. A qualitative analysis of predictors was performed with a focus on types of sexual regimes and level of human development.

**Results:**

One hundred thirty-five studies from 41 countries were included in the systematic review. The types of predictors varied according to the location of the study, the type of sexual regime and the level of gender inequality in that country/region. Consistently significant risk factors of female sexual dysfunction were: poor physical health, poor mental health, stress, abortion, genitourinary problems, female genital mutilation, relationship dissatisfaction, sexual abuse, and being religious. Consistently significant protective factors included: older age at marriage, exercising, daily affection, intimate communication, having a positive body image, and sex education. Some factors however had an unclear effect: age, education, employment, parity, being in a relationship, frequency of sexual intercourse, race, alcohol consumption, smoking and masturbation.

**Conclusions:**

The sexual and reproductive lives of women are highly impacted by female sexual dysfunction, and a number of biological, psychological and social factors play a role in the prevalence of sexual dysfunction. Healthcare professionals who work with women should be aware of the many risk factors for reproductive-age women. Future prevention strategies should aim to address modifiable factors, e.g. physical activity and access to sex education; international efforts in empowering women should continue.

**Electronic supplementary material:**

The online version of this article (10.1186/s12905-018-0602-4) contains supplementary material, which is available to authorized users.

## Background

Female sexual dysfunction affects 41% of reproductive-age women worldwide, making it a highly prevalent medical issue [1]. According to the Diagnostic and Statistical Manual for Mental Disorders (5th edition, 2013), female sexual dysfunction entails the following disorders: sexual interest/arousal disorder, female orgasmic disorder and genitopelvic pain/penetration disorder [2].

Sexual dysfunction has a biopsychosocial etiology, i.e. the origin of the dysfunction may stem from a biological or organic condition, a psychological condition and/or a social condition [3]. At the level of the individual, doctors aim to determine the etiology of the dysfunction and treat it accordingly. At the level of the population, however, researchers aim to predict which factors might put one population at risk over another population. Identifying these predictors and their effect (whether protective or risk-inducing) may aid health professionals to better detect and potentially prevent sexual problems from arising.

Past literature reviews have identified a number of similar biological, psychological and social predictors of female sexual dysfunction across different populations. In a 1990 systematic review on sexual dysfunction in both men and women, age, education, socio-economic status, and marital status were found to have an influence on male and female sexual dysfunction [4]. West et al.’s 2004 systematic review on female sexual dysfunction uncovered further predictors such as physical health (both observed and perceived), psychological health, race/ethnicity, number of premarital partners, religion, sexual orientation, communication with partner and attitude towards sexuality [5].

Predictors of sexual dysfunction are numerous, and various approaches can be used to classify and assess them. The gold standard in epidemiological research is to identify the exact effect sizes of predictors, that is, the quantitative effect of a specific risk factor or protective factor in a population expressed as a measure of relative and/or attributable risk. Such quantitative analyses require a certain degree of homogeneity in the observed population as well as in the measurement of the construct of interest, and therefore often focus on a limited number of predictors. In this analysis, however, the aim was not to quantify the magnitude of the effect of a single predictor but to uncover the breadth of predictors in heterogeneous populations around the globe and to identify possible trends. In order to provide a more structured analysis of the multifaceted risk factors and protective factors in these populations, the predictors of female sexual dysfunction were examined using paradigms which focused on gender inequality.

Two global studies, in particular, have shed some light on the association between female sexual function and gender inequality. In 2004, researchers from the Global Study of Sexual Attitudes and Behaviors (GSSAB) began publishing their results on a survey of 27,500 men and women in 29 countries [6]. In the wealth of data, they identified common gender-based trends of sexual attitudes in behaviors across the 29 surveyed countries [7]. The participants’ subjective responses on four components of sexual health (satisfaction with sexual functioning, physical pleasure, emotional pleasure, and importance of sex) revealed three clusters or so-called “sexual regimes.” A gender-equal sexual regime was found in typically Western countries. One type of male-centered sexual regime was identified in a mixed group of countries, and a second type of male-centered sexual regime was seen in predominantly Asian countries. Satisfaction with sexual functioning was one of four components composing the sexual well being score. A closer look at the responses in each country revealed that women in gender-equal regimes rated their satisfaction with sexual functioning at 64.4–91.1% [median 78.1%], while women in the mixed male-centered and the Asian male-centered regimes rated their satisfaction with sexual functioning at 44.5–82.1% [median 56.7%] and 39.7–61.3% [median 45.5%], respectively. Similar trends in women’s responses were also seen in the other three components of the sexual well being score. In all three clusters/regimes, women had consistently lower scores than men in terms of their sexual well being. However, differences were greater between men and women in the two male-centered regimes.

A 2016 systematic review and meta-analysis assessed the prevalence rate of female sexual dysfunction in 215,740 reproductive-age women worldwide and found the 41% of these women report some form of female sexual dysfunction [1]. A meta-regression of the collected data showed a positive correlation between the prevalence of female sexual dysfunction and the level of gender inequality in a country (Gender Inequality Index from the United Nations Development Program) [1, 8]. Further stratification of these results by world region illustrated that more developed regions (e.g. Europe and North America) typically had rates of female sexual dysfunction below 40%, whereas developing regions such as the Middle East and Africa had rates as high as 62%. The meta-analysis also stratified the prevalence rates according to sexual regimes, as identified by the GSSAB research group. While the overall prevalence rate of female sexual dysfunction was not significantly different between the three regimes, there were in fact significantly lower rates of pain disorders, orgasm disorder and lubrication difficulties in the gender-equal regime compared to the mixed and Asian male-centered sexual regimes. The results of these two large-scale studies cannot show causality between sexual dysfunction and gender inequality, but they do underline the importance of examining sexual health outcomes in terms of the level of gender inequality in a society.

With the rise in publications on female sexual dysfunction [9], an updated summary of the predictors of female sexual dysfunction is needed. The following qualitative analysis and its narrative synthesis will summarize the risk and protective factors related to female sexual dysfunction among reproductive-age women in multiple countries and simultaneously shed further light on the aspect of gender inequality.

## Methods

### Protocol and registration

The methods for this systematic literature search have been developed according to the recommendations from the Preferred Reporting Items for Systematic Reviews and Meta-Analyses (PRISMA) statements [10]. This protocol has been registered with the International Prospective Register of Systematic Reviews (PROSPERO): CRD42014009526 and is available in published form [11].

### Search strategy and selection criteria

Data for this review were identified by searches of Medline, Embase, PsycINFO, Web of Science and other relevant databases, using the terms “sexual dysfunction”, “female”, and “epidemiology”. Searches were limited to studies of humans, to the English language, and to the time frame January 1, 2000 until July 10, 2014.

The search was performed by an experienced medical research librarian. All titles and abstracts were screened for their relevance. If there was any uncertainty about an abstract’s relevance at this stage, the article remained included until the full text was reviewed. Articles identified through hand searches were considered for inclusion based on their titles.

A standard form was designed and used to evaluate the full-text publications for inclusion. Two investigators independently assessed each publication for eligibility and compared their results. If there was a discrepancy in their assessment, a final decision was taken based on discussions with a third reviewer. For multiple publications based on a single study, the most current and/or inclusive study was selected. A second hand search was performed using the reference lists of all included articles.

Cross-sectional, cohort, and case-control studies were eligible for this systematic review. Validation studies, reviews, reports, and commentaries were not included. Clinical populations or populations of women who were surveyed for a particular disease or illness were excluded, as the purpose of this systematic review was to capture the prevalence and risk factors present in the general population. Studies that addressed FSD in infertile women or couples and studies that examined spouses and partners of men with erectile disorder were also excluded.

The research question focused on reproductive-age women in the general population. Any studies that focused primarily on menopausal, postmenopausal, pregnant, or lactating women were excluded. Because several epidemiologic studies covered a broad age range of women, a numeric cutoff was used for the studies that did not specify which women were of reproductive age. Studies were included if (i) all women surveyed were described as premenopausal, (ii) the age range of the participants was between menarche and 49 years, or (iii) data on women no older than 49 years could be extracted from the entire population.

**Fig. 1:**
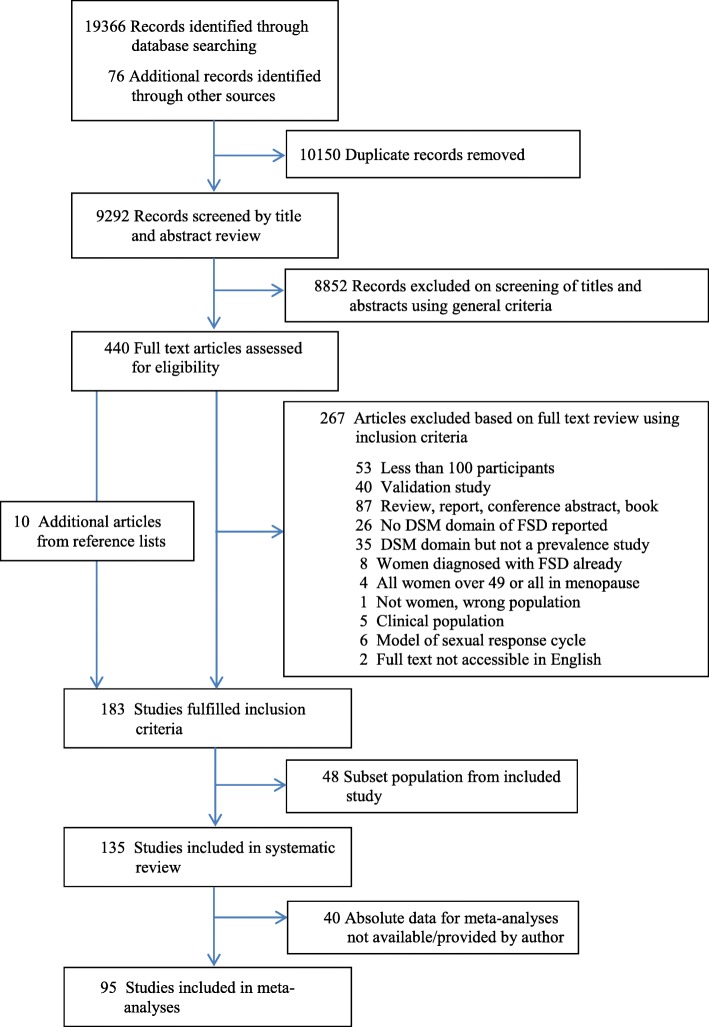
PRISMA flow chart showing number of citations retrieved from a systematic literature search in multiple databases

Further details regarding the search strategy, search terms, the assessment of bias, and the meta-analytical prevalence of female sexual dysfunction have been published elsewhere or may be requested from the corresponding author [1]. The PRISMA flow chart of the 135 studies included in this systematic review can be seen in Fig. 1.

### Data collection

Data were extracted from the included studies using an electronic data extraction form created in Microsoft Access. The extraction form was pre-designed and pilot-tested. A pilot test was performed with 20 randomly selected publications on the prevalence of female sexual dysfunction. Based on the results of the pilot test, the form was revised by the investigators.

Analyses of odds ratios and correlations within the included studies were examined in order to determine which predictors proved to be significant. Significant predictors (*P* < 0.05) were listed according to the following domains: overall female sexual dysfunction, hypoactive sexual desire disorder, female sexual arousal disorder, lubrication difficulties, female orgasmic disorder, and pain disorders. Where possible, information on significant predictors was taken from multivariate analyses; otherwise data from univariate analyses were extracted.

Once all data were extracted from the included publications, the data were examined and verified by a second author. Discrepancies in data entry were documented, discussed and revised accordingly.

### Qualitative analysis

A summary table describing each publication and its respective, significant risk/protective factors was created. Non-significant risk factors were also listed. The predictors extracted from the publications were then stratified using two different schemes: 1) the type of sexual regime and 2) the level of gender inequality/human development. For both schemes, similar risk factors/protective factors were grouped together, e.g. the term “relationship dissatisfaction” was used to represent terms such as “dissatisfied in marriage” and “poor relationship with husband;” the term “partner” was used instead of “husband” or “spouse.”

The type of sexual regime was based on results from the Global Study of Sexual Attitudes and Behaviors (GSSAB) which surveyed 27,500 men and women in 29 countries [7]. Using clustered data on sexual attitudes, satisfaction, behaviors, as well as prevalence rates of sexual dysfunction, Laumann et al. identified three types of sexual regimes worldwide: a gender-equal regime, a mixed male-centered regime, and an Asian sexual regime. The gender-equal sexual regime consisted primarily of Western/European nations (Austria, Belgium, France, Germany, Spain, Sweden, the United Kingdom, Mexico, Australia, Canada, New Zealand, South Africa, and the United States). The mixed male-centered sexual regime included Mediterranean countries (Algeria, Egypt, Israel, Italy, Morocco, and Turkey) as well as Korea, Malaysia, the Philippines, and Singapore. The third cluster, also considered male-centered, entailed only Asian countries: China, Indonesia, Japan, Taiwan, and Thailand (see Table 1).

**Table 1:** GSSAB sexual regimes according to Laumann et al. [7]

Countries with a gender-equal sexual regime	Countries with a male-centered sexual regime	
	mixed	Asian
Austria, Belgium, France, Germany, Spain, Sweden, the United Kingdom, Mexico, Australia, Canada, New Zealand, South Africa, and the United States	Algeria, Egypt, Israel, Italy, Morocco, Turkey, Korea, Malaysia, the Philippines, and Singapore	China, Indonesia, Japan, Taiwan, and Thailand

The significant predictors of female sexual dysfunction were stratified according to type of sexual regime and presented in a Venn diagram or in narrative form. For the individual domains of female sexual dysfunction (desire disorder, arousal disorder, lubrication difficulties, orgasm disorder and pain disorder), a narrative synthesis of the results was given.

For the level of human development, the Gender Inequality Index (GII), created by the United Nations Development Program, was used. The GII is based on current (ranging from 2010 to 2015) rates of maternal mortality, adolescent birth, women’s secondary education, women’s political involvement, and labor force participation [8]. Using these data, each country is given a GII value between 0 and 1: the higher the value, the greater the inequality between men and women. Based on the GII value, countries are sorted into quartiles with the following human development groups: very high human development, high human development, medium human development, and low human development. A link to the GII and the human development groups can be found here: http://hdr.undp.org/en/composite/GII .

The significant risk factors of female sexual dysfunction were stratified according to level of human development and illustrated in the form of word clouds. Due to a large number of highly-specific medical conditions addressed in these 135 publications, only the significant risk factors which were identified in at least two separate studies were included in the word cloud. This allowed for better comparison across the levels of human development. Furthermore, the word clouds were designed to be sensitive to the number of publications in which a certain risk factor had been identified, i.e. a risk factor which was identified in four publications would be presented in a larger font in the word cloud than a risk factor identified in only two publications. For the individual domains of female sexual dysfunction (desire disorder, arousal disorder, lubrication difficulties, orgasm disorder and pain disorder), a narrative synthesis of the results was provided.

## Results

### Eligible studies from the systematic literature search

The systematic literature search resulted in 135 eligible studies from 41 countries. Of the 135 studies, 97 publications (72%) from 34 countries analyzed predictors of female sexual dysfunction in populations of reproductive-age women [12–109]. A complete listing of both significant and non-significant predictors for each publication can be found in the supplementary material online [see Additional file 1]. Ninety-four publications from 33 countries reported significant predictors and were the basis for the following qualitative analyses (see Table 2).

**Table 2:** Characteristics of studies

Categorical variable	N (%)
World region	94
Europe	31 (33.0)
Non-Europe West^a^	14 (14.9)
Asia	19 (20.2)
Central and South America	10 (10.6)
Africa	9 (9.6)
Middle East	11 (11.7)
Sexual regime	56
gender-equal	27 (48.2)
male-centered	19 (33.9)
Asian (male-centered)	10 (17.9)
Human development group	92
very high	43 (46.7)
high	35 (38.0)
medium	11 (12.0)
low	3 (3.3)

^a^USA, Canada, South Africa, New Zealand, Australia

### Significant predictors of female sexual dysfunction

A summary of the significant predictors of female sexual dysfunction and each of its domains can be found in Table 3 Significant risk factors which were consistent in all domains of female sexual dysfunction were: poor physical health, poor mental health, poor partner health, partner unemployment, low education of partner, stress, abortion, menopause, genitourinary problems, female genital mutilation, relationship dissatisfaction, sexual dysfunction of partner, sexual abuse, and being religious. Factors which consistently had a significant, protective effect across all domains were: older age at marriage, exercising, daily affection, intimate communication, having a positive body image, sex education and finding sex to be “important.” For some factors, mixed results were reported in the studies, and a clear tendency (whether risk-inducing or protective) could not be found: age, education, employment, parity, being in a relationship, frequency of sexual intercourse, race, alcohol consumption, smoking and masturbation.

**Table 3:** Summary of predictors for female sexual dysfunction and its domains

	Risk factor	Unclear effect	Protective factor
Female Sexual Dysfunction	Demographic: unemployment, unemployment of partner, low education of partner, low SES, illiteracy, economic hardship, restrictive upbringing, sharing a bedroom with family members. Health and wellbeing: poor physical health, poor perceived health, poor mental health, low life satisfaction, poor quality of life, poor social relationships, environment with limited opportunities, chronic illness, heart disease, obesity, physical disability in previous year, depression, anxiety, taking antidepressants, dieting, alcohol, smoking, sleeping problems, polypharmacy. OBGYN: high number of births, ever pregnant, use of IUD, cervical erosion, late debut menarche, abnormal menstrual pattern, female genital mutilation, guilt about abortions, difficult delivery, menopause, urinary incontinence, endometriosis, yeast infection, gynecological surgery, genitourinary problems, pelvic inflammatory disease, hysterectomy, STI. Partner: poor partner health, partner smokes, older partner, partner has SD, relationship dissatisfaction, arranged marriage, polygamous relationship, living separately from partner, long duration of relationship. Sexual life: dissatisfaction with sex life, no / too little foreplay, no genital contact without intercourse (past month), ≥10 lifetime sexual partners, negative attitude toward sex, difficulty talking to partner about sex, not competent at first intercourse, bisexual preferences, homosexual preferences, non-sensuality, sexual abuse, sexual harassment, rape, dissatisfaction with partner’s penis size	age, level of education (high/low), level of income (high/low), residence (rural/urban), masturbation, use of contraceptives, use of HRT, being in a relationship/marriage, parity (having children/not having children), race	older age at marriage, faithful partner, access to private health care, emotional intelligence, frequent communication with partner, intimate communication, only 1 current sexual partner, pregnancy in last year, steady relationship without cohabitation, higher frequency of intercourse, church attendance, sex education, “sex is important”
Desire Disorder	Demographic: unemployment of partner, low education of partner, low SES, being religious, urban living, having young children, sharing a bedroom with family. Health and wellbeing: poor physical health, poor mental health, low life satisfaction, chronic illness, breast cancer, heart disease, diabetes, thyroid problems, hypertension, depression, anxiety, post-traumatic stress disorder (PTSD), drug addiction, habitualized negative thinking about oneself, dissatisfaction about how housework is done. OBGYN: late debut menarche, abnormal menstrual pattern, STI, female genital mutilation, tubal ligation, cervical erosion, ever pregnant, fear of pregnancy, birth in past year, menopause, urinary incontinence, genitourinary problems, hysterectomy, hormonal contraceptives, low hormones, multiparity. Partner: partner has SD, relationship dissatisfaction, internal stress with partner, habitualized negative thinking about partner, being widowed, long duration of relationship, married more than once. Sexual life: non-sensuality, sexual abuse, childhood sexual abuse, no / too little foreplay, low foreplay enjoyment, low sexual satisfaction, unidirectional coital initiation	age, level of education (high/low), level of income (high/low), employment (unemployed/full-time), masturbation, being in a relationship/marriage, race, frequency of intercourse	older age at marriage, moderate alcohol consumption, smoking, spontaneous sexual initiation, varied sexual repertoire, exercising, non-exclusive relationship, liberal attitudes towards sex, good communication with partner, intimate communication, early sexual debut, having >1 lifetime sexual partner, daily affection, currently pregnant, imbalance of commitment (woman more committed than man), sex education, “sex is important”
Arousal Disorder	Demographic: unemployment of partner, low education of partner, low SES, being religious. Health and wellbeing: poor physical health, poor mental health, chronic illness, arthritis, thyroid problems, irritable bowel, anxiety, depression, polypharmacy, physical abuse. OBGYN: urinary incontinence, genitourinary problems, menopause, hormonal contraceptives, fear of pregnancy. Partner: partner has SD, partner has low desire, relationship dissatisfaction, internal stress with partner, polygamous relationship, long duration of relationship. Sexual life: “sex is dirty”, no/too little foreplay, low foreplay enjoyment, high acceptance for pornography, liberal sex values, unidirectional coital initiation	age, level of education (high/low), employment (unemployed/full-time), being in a relationship/marriage, race	older age at marriage, divorced/widowed/separated, emotional intelligence, exercising, intimate communication, positive body image, higher frequency of intercourse, use of HRT, daily affection, “sex is important”
Lubrication Difficulties	Demographic: older age, unemployment, unemployment of partner, low education of partner, low SES, economic hardship, sharing a bedroom with family, manual laborer. Health and wellbeing: poor physical health, poor perceived health, poor mental health, chronic illness, anxiety, seeking medical help, physical abuse. OBGYN: abnormal menstrual pattern, late debut menarche, cervical erosion, infertility, urinary incontinence, STI, menopause. Partner: partner has SD, relationship dissatisfaction, long duration of relationship, partner is unattractive. Sexual life: masturbation, higher frequency of intercourse, “sex is dirty”, knowledge of clitoris	level of income (high/low), level of education (low/high), being in a relationship/marriage	older age at marriage, faithful partner, intimate communication, sex education, “sex is important”
Orgasm Disorder	Demographic: unemployment, unemployment of partner, low education of partner, urban living, sharing a bedroom with family, being religious, job insecurity, low SES, manual laborer. Health and wellbeing: poor physical health, poor mental health, chronic illness, smoking, alcohol, stress/anxiety, feelings of guilt, arthritis, thyroid problems, depression, critical life event, seeking medical help. OBGYN: late debut menarche, abnormal menstrual pattern, cervical erosion, STI, urinary incontinence, multiparity, abortion, fear of pregnancy, menopause. Partner: partner has SD, relationship dissatisfaction, partner is unattractive, polygamous relationship, married more than once, long duration of marriage. Sexual life: low foreplay enjoyment, masturbation, knowledge of clitoris, non-sensuality, “sex is a duty”, anti-masculinity, sexual embarrassment, rape by partner, no /too little foreplay, never/unsure if ever had orgasm, unidirectional coital initiation, sexual dissatisfaction, absence of sexual pleasure, unsatisfied with thickness/size of partner’s penis	age, level of education (low/high), level of income (high/low), being in a relationship/marriage, race, frequency of intercourse	older age at marriage, faithful partner, exercising, good communication with partner, intimate communication, satisfactory relationship with partner, use of contraceptives, daily affection, being divorced, married less than 5 years, sex education, “sex is important”
Pain Disorder	Demographic: unemployment, working overtime, unemployment of partner, low education of partner, urban living, sharing a bedroom with family, being religious, low SES. Health and wellbeing: poor physical health, poor perceived health, poor mental health, chronic illness, lung disease, arthritis, lower back pain, anxiety, exhaustion, seeking medical help, colitis, heavy lifting, constipation. OBGYN: late debut menarche, abnormal menstrual pattern, menopause, abortion, infertility, ever pregnant, early (< 15 years old) sexual debut, use of IUD, hormonal contraceptives, STI, chronic urinary tract infections, urinary incontinence, genitourinary problems, cervical erosion, pelvic organ prolapse, pelvic inflammation. Partner: partner smokes, relationship dissatisfaction, planning more children. Sexual life: masturbation, “sex is dirty”, varied sexual practices, sexual dissatisfaction, non-sensuality	age, level of education (high/low), level of income (high/low), being in a relationship/marriage, mode of delivery, parity (having children/not having children), race, frequency of intercourse	older age at marriage, at least 4 years of regular intercourse, positive body image, liberal attitudes towards sex, currently pregnant, sex education, “sex is important”

*SD* sexual dysfunction, *SES* socio-economic status, *IUD* intrauterine device, *HRT* hormone replacement therapy, *STI* sexually transmitted infection

### Significant predictors of female sexual dysfunction: Type of sexual regime

**Fig. 2:**
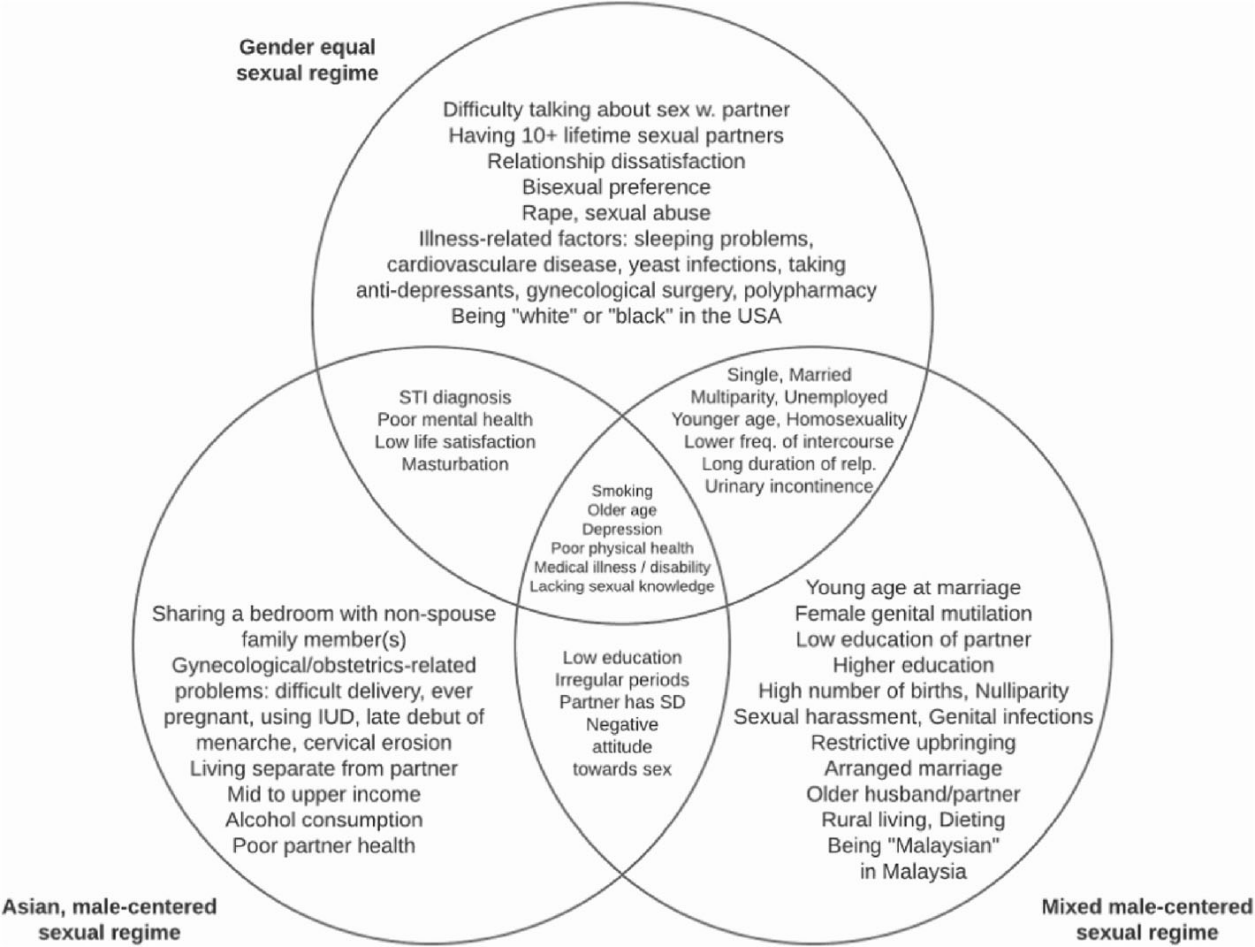
Venn diagram illustrating shared and unique risk factors for each sexual regime (*n* = 56)

Using the classification system from the GSSAB, the significant predictors extracted from the 94 publications were then analyzed based on the type of sexual regime in the given country (see Table 1 in Methods). The GSSAB data covered 29 countries, 15 of which were represented among the 94 publications. Thirty-eight publications could not be categorized, because the country of study had not been investigated in the GSSAB. However, 56 publications could be categorized into the following types of sexual regimes: gender-equal regimes (*n* = 27), male-centered regimes (*n* = 19), and Asian, male-centered sexual regimes (*n* = 10). Figure 2 illustrates the significant risk factors for female sexual dysfunction, shared among women living in various sexual regimes.

Independent of the type of sexual regime, a lack of sexual knowledge, medical illness, poor physical health, older age, depression and smoking were found to be common significant risk factors for female sexual dysfunction. Some risk factors were however unique to the individual regimes. The studies performed in countries with gender-equal sexual regimes (*n* = 27) reported risk factors which are associated a) with illnesses in Western lifestyle: cardiovascular disease, taking anti-depressants, sleeping problems, and polypharmacy or b) with sexual intimacy: difficulty talking with partner about sex, more than 10 sexual partners, relationship dissatisfaction, bisexual preference, and sexual abuse/rape. Studies performed in the mixed male-centered sexual regime (*n* = 19) indicated risk factors primarily associated with early partnership and reproduction: young age at marriage, older partner, arranged marriage, high number of births and nulliparity. Other risk factors unique to this regime were female genital mutilation, restrictive upbringing, rural living and dieting. Although there were not very many studies in Asian sexual regimes (*n* = 10), there was a trend in partner-related factors: sharing bedroom with non-spouse family member, living separate from partner, and poor partner health. Two significant risk factors which surfaced in Asian studies were also mid to upper income as well as alcohol consumption. Some of the significant protective factors noted in the various regimes included: higher frequency of intercourse (gender-equal and male-centered), use of contraceptives as well as sex education (male-centered), and frequent communication with partner (Asian).

### Significant predictors in the domains of female sexual dysfunction: Type of sexual regime

Risk factors for desire disorder were multitudinous but rather similar across all regimes (socio-economic difficulties, relationship difficulties, physical and mental health issues, etc.). The protective factors for desire disorders in the regimes were however unique. In gender-equal regimes, smoking and alcohol consumption had a protective effect, as well as spontaneous sexual initiation, masturbation, being in a non-exclusive relationship and having an imbalance of commitment in a relationship (woman more committed than man). For the mixed male-centered regime, alcohol consumption had a protective effect, as well as spontaneous sexual initiation, a varied sexual repertoire and sex education. Finally, for Asian male-centered sexual regimes, having a liberal attitude towards sex and being pregnant were protective factors for desire disorder.

Protective factors for arousal disorder in gender-equal regimes were: higher education, emotional intelligence, never married /widowed /divorced/separated, middle age (30–49), and using hormone replacement therapy. In contrast, being single was a risk factor for arousal disorder in the mixed male-centered regime and higher education was found to be a risk factor in Asian countries. Notably, having liberal sex values and a high acceptance for pornography were two further risk factors for arousal disorder in the Asian male-centered sexual regime.

Older age was a risk factor in mixed male-centered sexual regimes and gender-equal sexual regimes; relationship dissatisfaction was unique to the gender-equal sexual regime while being single was unique to the male-centered sexual regime. No protective factors for lubrication difficulties could be identified in the studies.

Protective factors of orgasm disorder across all regimes are worth highlighting: age group 30–40 (gender-equal), finding sex important (gender-equal and Asian male-centered), using contraceptives and being unmarried (mixed male-centered). With orgasmic disorder, there were more risk factors associated with the partner in both gender-equal and Asian regimes: relationship dissatisfaction, being unsatisfied with size/thickness of partner’s penis, low foreplay enjoyment, unidirectional coital initiation, no daily affection, unattractive partner, adulterous partner, and partner has sexual dysfunction. Other risk factors which were unique to the Asian male-centered sexual regime were masturbation in the past 12 months and knowledge of the clitoris.

Lastly, for pain disorder, older age was found to be a risk factor and a protective factor for all three sexual regimes. A complete list of risk factors and protective factors for all domains according to sexual regime can be found in the supplementary material online [see Additional file 2].

### Significant predictors of female sexual dysfunction: Level of human development

**Fig. 3:**
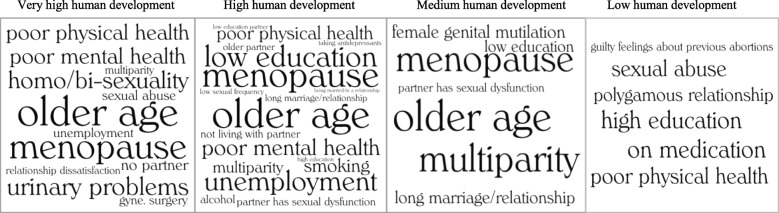
Word clouds of the significant risk factors for female sexual dysfunction according to the level of human development (*n* = 94)

Using the GII quartiles, the predictors were assessed in terms of the level of human development (HD) in the given country. The GII data cover all countries which were represented in our data. Two multinational studies among the 94 publications surveyed women in countries with different GII quartiles, thus they could not be included in the current analysis [27, 86]. However, 92 studies could be sorted into the following quartiles: very high human development (*n* = 43), high human development (*n* = 35), medium human development (*n* = 11), and low human development (*n =* 3). Figure 3 uses word clouds to illustrate the frequency and variety of significant risk factors for female sexual dysfunction, stratified by the level of human development.

The risk factors illustrated in the word clouds have commonalities and differences. In very high, high and medium HD groups, menopause and older age were risk factors in the observed studies. Although this analysis focused on reproductive-age women, some studies with broad age ranges also included women going through menopause. These risk factors were not apparent in studies in countries from the low HD group (Nigeria, Kenya, Uganda and Ethiopia). Contradictory risk factors were evident, e.g. low vs. high education. Risk factors related to sexual oppression were exposed through the stratification process. Homosexual and bisexual women are at a greater risk for sexual dysfunction in the very high HD group, while women who are in polygamous relationships and those who have gone through female genital mutilation are at risk in medium and low HD groups. Sexual abuse was a risk factor in both very high and low HD groups.

Protective factors in studies conducted in countries with very high HD were: good overall health, higher education, positive body image, exercising, masturbating, moderate alcohol consumption, smoking, higher number of lifetime partners, church attendance, intimate communication, and the use of hormone replacement therapy. Studies conducted in countries with high HD revealed similar protective factors: good overall health, higher education, moderate alcohol consumption, and good communication with partner. Other significant protective factors in the high HD group were: using contraceptives, having sex education, finding sex to be “important,” an older age at marriage, spontaneous rather than unidirectional sexual initiation, and a varied sexual repertoire. Studies in the medium and low HD groups yielded only a few significant, protective factors; these were higher income, having some education, and the use of hormone replacement therapy.

### Significant predictors in the domains of female sexual dysfunction: Level of human development

Risk factors and protective factors in each domain of female sexual dysfunction were analyzed, but the findings did not provide further insight beyond that which had already been exposed through the previous analyses. A list of the significant risk factors for all domains according to level of human development can be found in the supplementary material online [see Additional file 3].

## Discussion

### Indication of imbalance

Through the increase in population-based studies around the globe, the number and variety of predictors have increased as well. This is the first systematic review to address significant predictors of female sexual dysfunction for each domain of sexual dysfunction. Due to the heterogeneous populations and the fact that studies assessed different domains of female sexual dysfunction, a wide variety of predictors could be identified among the 94 international studies. Factors which consistently had a significant, protective effect across all domains were: older age at marriage, exercising, good overall health, daily intimacy and relationship satisfaction, having a positive body image, sex education and finding sex to be “important.” Risk factors were frequently related to both physical and mental health of women. Other significant factors such as age, partnership, and parity showed mixed protective and risk effects in the populations and within the domains of sexual dysfunction.

Further stratification of these predictors was essential to this analysis. Because there were lower meta-analytical prevalence rates in gender equal sexual regimes and because of the correlation between high female sexual dysfunction rates and high gender inequality [1], the risk factors and protective factors were examined through paradigms of gender inequality in order to better understand trends in predictors of female sexual dysfunction.

### Stratification reveals research gap

Trends of predictors could be identified once the studies were stratified according to type of sexual regime (56 studies). In gender-equal sexual regimes (Western/European countries), risk factors tended to be related to chronic illness and mental health, as well as to quality of life factors. In the Asian male-centered sexual regime, risk factors were related to gynecological health but also to family and partner – more so than in the other two regimes. In the mixed male-centered sexual regimes (primarily Arab and African countries), risk factors including female genital mutilation, restrictive upbringing and high number of births point towards underlying challenges in women’s rights and women’s reproductive health.

Risk factors such as older age, poor health, and relationship dissatisfaction were found in all human development groups, regardless of the level of gender inequality (*n* = 92). This stratification also revealed that far fewer studies can be found in countries with high to very high gender human development vs. those with medium to low human development (78 vs. 14). Studies from countries with low/medium human development accounted for less than 15% of all studies in this analysis, indicating a lack of research in these countries and limited knowledge concerning the predictors of female sexual dysfunction in these populations.

### Risk factor or protective factor?

Former systematic reviews on sexual dysfunction primarily included studies from Western nations, i.e. the predictors of sexual dysfunction were based on gender-equal sexual regimes/countries with high development. With the growing number of studies in developing nations and Asian countries, it is evident that not all risk factors and protective factors are universal. In a large US study, education was identified as a protective factor against sexually distressing problems [87]. In studies from Iran and Jordan, young women who are educated and have gainful employment are less likely to show symptoms of sexual dysfunction [12, 84, 99]. However, several studies from China have shown that young women who have higher education were more likely to report sexual dysfunction [62, 109, 110]. Through higher education, these women gain increased awareness of their sexual needs and rights, and such women tend to feel more disappointed with their marital and sexual relationships, which may lead to poor sexual functioning [89, 111]. Similarly, while increased frequency of sexual intercourse was found to have a protective effect in most cultures, some studies in traditional cultures showed that frequent sex might be demanded by the partner and is therefore a risk factor for sexual dysfunction in these women [62, 73].

Some predictors showed variation within the domains. For example, female sexual dysfunction has generally been shown to be age-related [112]. Older age tends to be a risk factor for all domains except for pain disorder(s), where it is shown to have a protective effect. Other studies showed a U-shaped prevalence of sexual dysfunction, with younger and older women being most affected [113]. Women in their 30s may show fewer symptoms of dysfunction as they learn more about their preferences and become more comfortable accepting and expressing their sexuality [103]. Similar variation in the effect (whether positive or negative) was found for employment, income, partnership status, and parity.

Laumann et al. established two different male-centered sexual regimes, because the Asian sexual regime had clear differences in attitudes and behaviors when compared to Middle Eastern or African countries [7]. Risk factors such as high acceptance of pornography, masturbation, liberal sex values and knowledge of the clitoris were unique to Asian population studies. The authors explain that in these societies such women are considered non-traditional. Women who do not conform to traditional female roles in these societies may experience greater difficulties with their male partners [62].

Two further predictors which were shown to have a mixed effect on women’s sexual functioning were alcohol consumption and smoking. In the majority of studies, these factors did not have an effect on sexual functioning. However, selected studies suggest that these factors may be a mediating factor for improved sexual desire. In a Puerto Rican study, smoking was shown to have a significant protective effect in respect to desire disorder [21]. The authors describe that it may not be the act of smoking itself that is protective but rather the liberal lifestyle associated with women who smoke in that society. Similarly with alcohol consumption, the studies generally showed a non-significant effect, but three studies revealed a protective effect of moderate alcohol consumption [13, 21, 41]. In the three countries represented in the studies (Brazil, Denmark, Puerto Rico), the authors came to the conclusion that moderate alcohol consumption is associated with lower rates of desire disorder. As with smoking, the underlying component may not be the alcohol, but instead a less restrictive approach to “traditional” female roles.

### Challenges in preventing sexual dysfunction

Protective factors, regardless of population, regime, or level of development, were: sex education, exercise, older age at marriage, daily affection, intimate communication, having a positive body image, and finding sex to be “important.” Since several of these factors are modifiable, preventive measures could be taken to potentially avert the onset of female sexual dysfunction. However, these factors may be more complicated to address in some countries than in other countries, as they are closely entwined with culture.

Sex education has a significant protective effect [13, 61, 69], but sex education and reproductive health services in many countries tend to focus exclusively on married women, as it is culturally unacceptable for single women to have sexual relations. The needs of young, unmarried, sexually-active women may therefore go unaddressed [114].

Similarly, while exercise may seem like a reasonably modifiable risk factor for female sexual dysfunction, mobility, e.g. exercising, traveling and moving about in public spaces, can be challenging for women living in countries with high gender inequality. An international study of 70 countries revealed that women’s lack of autonomy and resources to move freely can result in mobility disability [115]. This means that women in countries with higher gender inequality may not be able to have healthy lifestyles, e.g. getting enough physical activity or traveling to the doctor to receive care.

Furthermore, male-dominated cultures, in which sexual behavior is oriented more towards reproduction, tend to suppress women’s sexual needs and pleasure and to discount the relational meaning of sex [7, 62]. Current practices in these cultures such as arranged marriages, marriages at a young age, polygamy and female genital mutilation are associated with significantly higher levels of sexual dysfunction in women [19, 38, 42, 44, 73].

Finally, women in conservative cultures women may also be too timid to express their needs or feel that it is socially unacceptable to discuss sexual problems with their partner [65, 76, 111]. While lower rates of sexual dysfunction are found in women who share intimate communication with their partner [67], this may be easier in some cultures than in others.

Challenges in improving women’s health are numerous. Gender inequality creates an additional barrier in terms of women’s sexual and reproductive health. It is for this reason that the World Health Organization has made it one of its Millennium Development Goals to promote gender equality and empower women [114]. Research on gender inequality takes a considerable amount of time, as changes in cultural patterns do not take place overnight. However, health studies have confirmed the association between gender inequality and women’s wellbeing. A recent ecological study from Stanford University on global HIV prevalence rates and the GII showed an overall positive correlation between the two variables (*r* = 0.525, *p* < 0.001) [116]. Furthermore, they were able to illustrate limited but compelling evidence that improvements in gender inequality can lead to the abatement of generalized HIV epidemics in countries with predominantly heterosexual transmission. Additionally, a study on reproductive health in 75 countries revealed that the empowerment of women was associated with the improvement of several health factors, including but not limited to fertility, maternal mortality, and low birth weight of the infant [117].

### Limitations of the study

The literature review aimed to collect all available data on the prevalence and predictors of female sexual dysfunction among reproductive-age women, globally. While this literature review and analysis on female sexual dysfunction covers studies from more countries than previous reviews, there were many countries which were not represented in this assessment (*n* = 161). This lack of data may be due to the fact that the search was limited to the English language or due to a lack of published research on female sexual dysfunction in these countries.

Due to the study design, a causal association between female sexual dysfunction and the reported predictors cannot be demonstrated, nor can the findings be extrapolated to the individual level. Furthermore, a quantitative analysis would be necessary in order to determine the exact effect sizes of the risk/protective factors.

Finally, while this analysis summarizes a wealth of available data on significant predictors, it simultaneously reveals a dearth of research on women’s sexual health. Factors which were not documented in this analysis are not irrelevant to sexual dysfunction, i.e. a factor which was not identified in a population/group/regime does not mean it is not pertinent to that population/group/regime. More in-depth research is needed, both quantitative and qualitative, in the field of women’s sexual health – particularly in regions with male-centered sexual regimes and high gender inequality.

## Conclusions

The sexual and reproductive lives of women are highly impacted by female sexual dysfunction, and a number of biological, psychological and social factors play a role in the prevalence of sexual dysfunction. Healthcare professionals who work with women should be aware of the many risk factors for reproductive-age women. Future prevention strategies should aim to address modifiable factors, e.g. physical activity and access to sex education; international efforts in empowering women should continue.

## Additional files

Additional file 1: Supplementary Material. Predictors by publication. (DOCX 64 kb)

Additional file 2: Supplementary Material. Significant factors stratified by sexual regime. (DOCX 22 kb)

Additional file 3: Supplementary Material. Significant risk factors stratified by level of human development. (DOCX 27 kb)
