# Supplementary material for: Predictors of female sexual dysfunction: a systematic review and qualitative analysis through gender inequality paradigms
Source: BMC Womens Health. 2018 Jun 22;18:108. doi: 10.1186/s12905-018-0602-4 (PMC6013982; doi:10.1186/s12905-018-0602-4)
Supplement: Supplementary file 1 — Supplementary Material. Predictors by publication. (DOCX 64 kb) [file 12905_2018_602_MOESM1_ESM.docx]

| **Supplementary Material**  **Predictors by Publication** | |  |  |  |
| --- | --- | --- | --- | --- |
| **Title** | **Author, year** | **Risk factor** | **Protective factor** | **Non-significant factors** |
| Prevalence of sexual dysfunctions and correlated conditions in a sample of Brazilian women—results of the Brazilian study on sexual behavior (BSSB) | Abdo et al., 2004 [12] | **FSD**: lower education **Desire:** aged 26 to 40, aged 41 or older, only high school education, only elementary education, race ("brown"). **Pain:** unmarried, only high school education, only elementary education. **Orgasm:** aged 41 or older, unmarried, only high school education, only elementary education. | **Pain**: aged 26 to 40, aged 41 or older, race (brown), orgasm: unmarried. **Desire**: unmarried. | **Desire:** race (black and other) pain: race (black, brown, other), depression, cardiopathies. **Orgasm**: race (black, brown, other) aged 26 to 40, separated/divorced /widowed, religion, non-heterosexual orientation, depression, cardiopathy, hypercholesterolemia. **Pain**: separated/divorced /widowed, race (black, brown, other), religion, non-heterosexual orientation, diabetes, cardiopathy, systematic arterial hypertension, hypercholesterolemia. |
| Hypoactive sexual desire disorder in a population-based study of Brazilian women: Associated factors classified according to their importance | Abdo et al., 2010 [13] | **Desire:** older age, widowed, low education, having children, unemployment, cardiovascular disease, diabetes, breast cancer, PTSD, hypertension, depression, low hormone levels, excessive use of medication (addiction), first sexual intercourse after age 21, only one significant sexual partner in lifetime, no foreplay or too little foreplay. | **Desire**: single or divorced, moderate alcohol consumption, sex education, spontaneous sexual initiation, varied sexual repertoire. |  |
| Factors associated with sexual dysfunction in Jordanian women and their sexual attitudes | Abu Ali et al., 2009 [14] | **FSD**: older age, obesity, higher education. |  | **FSD**: SES. |
| Sexual dysfunction among secondary school teachers in Ilorin, Nigeria | Adegunloye et al., 2010 [15] | **Orgasm**: being single. **Pain**: being married. |  |  |
| Sexual dysfunction in women: An overview of risk factors and prevalence in Indian women | Aggarwal et al., 2012 [16] | **FSD:** illiteracy, lower education level, chronic disease, menopause, pelvic inflammatory disease, endometriosis, 24-30 years, 38-42 years, older than 42 years |  | **FSD**: primary education, higher secondary education, graduate education, post graduate education, history of candida infection, <24 years,31-37 years. |
| Incidence of sexual dysfunction: A prospective survey in Ghanaian females | Amidu et al., 2010 [17] | **FSD**: alcohol, non-sensuality. **Pain (vaginismus):** increased with non-sensuality and infrequency and older age. **Orgasm**: non-sensuality. | **Aversion**: older age. | **FSD:** exercise, smoking, married, educational attainment, aged older than 36 years. |
| Sexual dysfunction among married couples living in Kumasi metropolis, Ghana | Amidu et al., 2011 [18] | **FSD**: long duration of marriage (13+ yrs). |  | **FSD**: smoking, alcohol, level of education, physical activity. |
| Survey of the prevalence of sexual dysfunctions in Kurdish women | Arasteh et al., 2013 [19] | **FSD**: older age. | **FSD**: older age at marriage. | **FSD:** rural or urban, level of education, employment status. |
| Prevalence and risk factors for low sexual function in women: A study of 1009 women in an outpatient clinic of a university hospital in Istanbul | Aslan et al., 2008 [20] | **FSD**: older age, low education (less than 8 yrs), menopause, depression, partner has SD. | **FSD**: using some form of contraceptive. | **FSD**: partner's age, duration of marriage, age at first sexual experience, smoking, alcohol, chronic disease, drugs, sexual abuse, HRT. |
| Relationship between loss of libido and signs and symptoms of depression in a sample of Puerto Rican middle-aged women | Avellanet et al. 2008 [21] | **Desire**: having symptoms of depression, genitourinary symptoms, older age, unemployment, having children, menopausal. | **Desire**: smoking, drinking alcohol, exercising 2x per week. | **Desire**: BMI, use of oral contraceptives. |
| Dyspareunia in Puerto Rican middle-aged women | Avellanet et al., 2009 [22] | **Pain**: low educational attainment, working status, with a partner, menopausal/HRT, urinary incontinence. | **Pain**: current employment. | **Pain**: use of oral contraceptives, current cigarette smoking, BMI, exercising at least 2x per week, any alcohol use. |
| Prevalence of female sexual dysfunction and related factors for under treatment in Bushehrian women of Iran | Bagherzadeh et al., 2010 [23] | **FSD**: low education, low education of husband, unemployment, unemployment of husband, low SES, smoking, husband smokes. **Desire**: urban, low education, low education of husband, unemployment of husband, low SES. **Arousal**: low education, low education of husband, unemployment, unemployment of husband, low SES. **Lubrication**: low education, low education of husband, unemployment, unemployment of husband, low SES. **Orgasm**: low education, low education of husband, unemployment, unemployment of husband, low SES, smoking. **Pain**: low education of husband, unemployment, unemployment of husband, low SES, husband smokes. |  | **FSD**: urban or rural, addiction of husband, husband drinks alcohol, husband drug consumption. **Desire**: employment, smoking, husband smoking, addiction of husband, husband drinks alcohol, husband drug consumption. **Arousal**: urban or rural, husband smoking, addiction of husband, husband drinks alcohol, husband drug consumption. **Lubrication**: urban or rural, husband smoking, addiction of husband, husband drinks alcohol, husband drug consumption. **Orgasm**: urban or rural, husband smoking, addiction of husband, husband drinks alcohol, husband drug consumption. **Pain**: urban or rural, level of education, smoking, addiction of husband, husband drinks alcohol, husband drug consumption. |
| Vulvar pain, sexual behavior and genital infections in a young population: A pilot study | Berglund et al., 2002 [24] | **Pain**: regular sexual intercourse before the age of 15 years or earlier, using oral contraceptives for more than 2 years. | **Pain:** More than 4 years of regular intercourse; coitus at least 4x a week. | **Pain:** STD; history of candida infection, chronic UTI. |
| The impairment of sexual function is less distressing for menopausal than for premenopausal women | Berra et al., 2010 [25] | **Desire**: older age. **Arousal**: older age. **Lubrication**: older age. **Orgasm**: older age. **Pain**: older age. |  |  |
| Sexual desire and sexual activity of men and women across their lifespans: Results from a representative German community study | Beutel et al., 2007 [26] | **Desire**: older age, lack of partnership, childhood sexual abuse |  | **Desire:** income, employment status. |
| Sexual dysfunction in middle-aged women: A multicenter Latin American study using the Female Sexual Function Index | Blumel et al., 2009 [27] | **FSD**: poor lubrication, older age, urinary incontinence, HRT, negative perception of own health, being married, partner has SD. | **FSD**: higher education, access to private health care, faithful partner. |  |
| Associations among everyday stress, critical life events,  and sexual problems | Bodenmann et al., 2006 [28] | **Desire**: internal stress (within couple). **Aversion**: internal stress. **Arousal**: poor relationship quality, internal stress. **Orgasm**: critical life event. |  | **Desire**: psychological symptoms, relp quality, external stress (job, outside life), critical life event. **Aversion**: psychological symptoms, relp quality, external stress, critical life event. **Arousal**: psychological symptoms, external stress, critical life event. **Orgasm**: psychological symptoms, relp quality, internal stress, external stress. **Pain**: psychological symptoms, relationship quality, internal stress, external stress, critical life event. |
| Effect of parity on sexual function - An identical twin study | Botros et al., 2006 [29] | **FSD**: multiparity, urinary incontinence. |  | **FSD**: age, menopause, BMI, smoking, hysterectomy, mode of delivery, weight of baby, episiotomy. |
| Recent and lifelong sexual dysfunction in a female UK population sample: Prevalence and risk factors | Burri and Spector, 2011 [30] | **FSD**: relationship dissatisfaction. **Arousal**: older age. **Lubrication**: older age, relationship dissatisfaction. **Orgasm**: older age, relationship dissatisfaction. **Pain**: relationship dissatisfaction, anxiety. | **FSD**: emotional intelligence. AD: emotional intelligence. | **FSD**: BMI, history of emotional abuse, history of sexual abuse, history of physical abuse, number of pregnancies, parity, smoking. |
| Sexual functioning and practices in a multi‐ethnic study of midlife women: Baseline results from SWAN | Cain et al., 2003 [31] | **Desire:** ethnicity (Chinese, Japanese), older age, never being married, paying for basics is hard, paying for basics is moderately hard. **Pain:** perimenopause, race (African American, Chinese) marital status (never married, widowed/separated/divorced). **Arousal:** race (African American, Hispanic, Chinese). | **Pain:** older age. **Arousal:** never married, widowed/separated/divorced, college graduate, more than college education. | **Desire:** African American, Hispanic, perimenopause, widowed/separated/divorced **Arousal:** perimenopause, less than high school education, more than high school education, paying for basics moderately hard, paying for basics hard, **pain:** race (Hispanic, Japanese) |
| Prevalence of sexual dysfunction in a cohort of middle-aged women: Influences of menopause and hormone replacement therapy | Castelo-Branco et al., 2003 [32] | **FSD:** partner has ED, hysterectomy, menopause, age > 49 years. | **FSD:** church attendance, higher education, good general health, HRT. | **FSD:** alcoholic husband, sexual abuse history, psychiatric complaints, only 1 sexual partner, stable couple, healthy husband. |
| The prevalence of female sexual dysfunction and potential risk factors that may impair sexual function in Turkish women | Cayan et al., 2004 [33] | **FSD**: older age, low education, unemployment, chronic disease, multiparity, menopause. |  | **FSD**: smoking history, age at marriage, pelvic surgery, contraceptive methods used. |
| Sexual dysfunctions and difficulties in Denmark: Prevalence and associated sociodemographic factors | Christensen et al., 2011 [34] | **FSD**: living in city with more than 100,000 inhabitants (urban living), more than 12 years of school attendance, high further education, economic hardship in the family, unmarried. **Lubrication**: non or semi-skilled worker, difficulties paying bills during the previous year. **Orgasm**: living in a city with more than 100,000 inhabitants (urban living), unmarried women. **Pain**: living in a city with more than 100,000 inhabitants (urban living). |  | **FSD**: towns with 10,000-99.999 inhabitants (rural living), current employment, difficulties paying bills in the previous year. **Lubrication**: living in a city with more than 100,000 inhabitants (urban living), towns with 10,000-99.999 inhabitants (rural living), years of school attendance, low or intermediate education, current employment, household incomes, marital status. **Orgasm**: years of school attendance, low intermediate or high education, current employment, economic hardship in the family. **Pain**: years of school attendance, low intermediate or high education, current employment, economic hardship in the family, household income, difficulties paying bills in the previous year, marital status. |
| Prevalence and incidence of prolonged and severe dyspareunia in women: Results from a population study | Danielsson et al., 2003 [35] | **Pain:** younger age (20-29, 30-39). |  | **Pain:** use of oral contraceptives. |
| Study of the prevalence of female sexual dysfunction in sexually active women 18-40 years of age in Medellin, Colombia | Echeverry et al., 2010 [36] | **FSD:** elementary education, high school education, not living together, depressive feelings, current use of antidepressives. |  | **FSD**: aged 31-40, economic status, marital status, former use of antidepressants, contraceptive use, type of contraceptive, irregular menstrual cycle, children. |
| Young Swedish women’s experience of pain and discomfort during sexual intercourse | Elmerstig et al., 2009 [37] |  |  | **Pain**: pain during first sexual intercourse, perception (positive/negative) of experience of first sexual intercourse. |
| The impact of female genital cutting on health of newly married women | Elnashar and Abdelhady, 2007 [38] | **FSD:** female genital mutilation. |  |  |
| Female sexual dysfunction in Lower Egypt | Elnashar et al., 2007 [39] | **FSD:** older age, secondary education, female genital mutilation, abnormal menstrual pattern, nulliparity, having 3 or more children (multiparity). |  | **FSD:** residency (rural or urban), type of family (extended or nuclear), employment status, duration of marriage, having other wives, mode of delivery, mode of contraceptive. |
| Female sexual dysfunction in urological patients: findings from a major metropolitan area in the USA | Elsamra et al., 2010 [40] | **FSD:** age, menopause, SSRI usage (selective serotonin reuptake inhibitors). |  | **FSD:** chronic medical disease, psychiatric illness, smoking, alcohol use, history of pelvic surgery, radiotherapy or malignancy. |
| Sexual desire in a nationally representative Danish population | Eplov et al., 2007 [41] | **Desire**: older age, low SES, sexual abuse, anxiety, emotional problems. | **Desire**: moderate alcohol consumption, exercising. | **Desire**: marital status, level of education, parity, self-perceived health status, diabetes. |
| Prevalence and risk factors for female sexual dysfunction among Turkish women attending a maternity and gynecology outpatient clinic | Erbil, 2011 [42] | **FSD:** younger age at marriage, higher number of births, higher number of children, housewife (unemployment), rural residence, low education, low education level of the husband, being brought up by parents with restrictive attitudes, having genital infections or symptoms, arranged marriage. | **FSD**: sex education. | **FSD:** age, duration of marriage, BMI, income, family type (large/small), mode of delivery, opinion on virginity (abstinence), chronic disease, depression, menopause. |
| Level of bother and treatment-seeking predictors among male and female in-patients with sexual problems: A hospital-based study | Evangelia et al. 2010 [43] | **FSD**: older age. |  |  |
| Sexual dysfunction among female patients of reproductive age in a hospital setting in Nigeria | Fajewonyomi et al., 2007 [44] | **FSD:** tertiary education higher education), history of sexual abuse, guilt feelings about previous abortions, medications, current illness, polygamous relationship. |  |  |
| Presence of a sexual problem may not affect women's satisfaction from their sexual function | Ferenidou et al., 2008 [45] | **Desire:** older age. **Arousal:** older age. **Lubrication:** older age. **Orgasm:** older age. |  | **Pain:** age |
| Pelvic pain and associated characteristics among women in Northern Mexico | Garcia-Perez et al., 2010 [46] | **Pain**: older age, age of sexual debut 10-14, cesarean birth, IUD use, history of STI /PID, history of colitis, chronic UTI. |  | **Pain**: marital status, having uterine fibroids, BMI, height. |
| Female sexual dysfunction in Iran: Study of prevalence and risk factors | Ghanbarzadeh et al., 2013 [47] | **Orgasm**: housewife or manual laborer. **Pain**: nulliparity, having 1-3 deliveries. | **Orgasm**: satisfactory relp with husband, being sexually active. | **Orgasm**: age, education duration of marriage, menopause status, method of contraception, chronic disease, use of medication. **Pain**: not reported. |
| Prevalence and potential risk factors of female sexual difficulties: An urban Iranian population-based study | Goshtasebi et al., 2009 [48] | **Desire**: older age, tubal ligation. **Arousal**: older age. **Pain**: low education. | **Arousal**: older age at marriage. **Orgasm**: using contraceptives. **Pain**: older age. | **Desire**: level of education, employment status, age at marriage, contraceptive method. **Arousal**: level of education, employment status, contraceptive method, genital surgery. **Lubrication**: age, education, employment status, age at marriage, contraceptive method, genital surgery. **Orgasm**: age, education, employment, age at marriage, genital surgery. **Pain**: employment, age at marriage, contraceptive method, genital surgery. |
| Prevalence and evaluation of sexual health problems - HSDD in Europe | Graziottin, 2007 [49] | **Desire**: older age |  |  |
| Prevalence and risk factors of female sexual dysfunction among healthcare personnel in Malaysia | Grewal et al., 2014 [50] | **Desire:** low frequency of intercourse, partner with ED, longer duration of marriage. |  |  |
| Self-reported frequency of feeling sexual desire among a representative sample of 18-49 year old men and women in Oslo, elicudated by epidemiological data | Hamilton et al., 2001[51] |  | **Desire**: masturbating in last 30 days. |  |
| Prevalence and characteristics of female sexual dysfunction in a sample of women from Upper Egypt | Hassanin et al., 2009 [52] | **FSD**: older age, multiparity, married more than 10 years, menopause, female genital mutilation. |  | **FSD**: level of education, use of contraceptive, urban or rural, age of husband. |
| Risk factors for female sexual dysfunction in the general populations: Exploring factors associated with low sexual function and sexual distress | Hayes et al., 2008 [53] | **Desire**: unemployment, sex is not important, in relationship for 20-29 years, dissatisfaction with partner. **Arousal**: perimenopausal, postmenopausal, depression, low education, sex is not important. **Orgasm:** sex is not important. | **Arousal**: middle age (30-49), taking hormone therapy. **Orgasm:** middle age (30-39). | **Desire**: age, menstrual cycle, HRT, depression, antidepressants, level of education, employment, forced into sexual activity, relationship status, communication. **Arousal**: HRT, antidepressants, employment, forced into sexual activity, relationship status, duration of partnership, communication, sexual satisfaction. **Orgasm**: menstrual cycle, HRT, depression, antidepressants, level of education, employment ,forced into sexual activity, relationship status, duration of partnership, communication, sexual satisfaction. |
| Prevalence of female sexual dysfunction symptoms and its relationship to quality of life: A Japanese female cohort-study | Hisasue et al., 2005 [54] | **FSD:** older age. **Desire:** older age. **Arousal**: older age. **Lubrication**: older age. **Orgasm**: older age. **Pain**: older age. |  |  |
| Prevalence and risk factors for female sexual dysfunction among Egyptian women | Ibrahim et al., 2013 [55] | **FSD:** older age, menopausal, married over 10 years, female genital mutilation, partner over 50 years old, partner has SD. | **FSD**: HRT | **FSD**: education, parity, smoking, chronic disease, rural or urban, mode of delivery, use of contraceptives. |
| Prevalence, risk factors, and predictors of female sexual dysfunction in a primary care setting: A survey finding | Ishak et al., 2010 [56] | **FSD**: having a medical illness, menopause, low frequency of intercourse. |  | **FSD**: age, husband’s age, duration of marriage, use of contraceptives. |
| Female sexual dysfunction: Prevalence and risk factors | Jaafarpour et al., 2013 [57] | **FSD:** older age, lower education, sexual intercourse less than 3x per week, having 3 or more children, having a husband age 40 or more, being married 10 years or more, unemployment. |  | **FSD**: contraceptive use, residency (rural or urban), smoking history. |
| Female sexual dysfunction: Facts and factors among gynecology outpatients | Jahan et al., 2012 [58] | **FSD**: multiparity, mental stress, partner has SD. |  | **FSD**: mode of delivery, abortion, contraceptive, history of STD, hypertension, pelvic surgery. |
| Risk factors for the individual domains of female sexual function | Jiann et al., 2009 [59] | **Desire**: parity, married 6-10 years (vs. <5 or >10), diabetes, poor relp with partner, no steady partner, partner has ED. **Arousal**: older age, poor relp with partner, partner has ED, partner has prem ejaculation, partner has low desire. **Lubrication**: older age, urinary incontinence, no steady partner, partner has ED. **Orgasm**: poor relp with partner, partner has ED, partner has prem ejaculation. **Pain**: younger age, urinary incontinence, no steady partner. | **Orgasm**: married 5 yrs or less | **Desire:** age, urinary incontinence, partner has PE, partner has low desire. **Arousal:** children, duration of marriage, diabetes, urinary incontinence. **Lubrication**: children, duration of marriage, diabetes, partner has PE, partner has low desire. **Orgasm**: age, children, diabetes, urinary incontinence, partner has low desire. **Pain**: children, duration of marriage, diabetes, partner has ED, partner has PE, partner has low desire. |
| Sexual motivation and the duration of partnership | Klusmann, 2002 [60] | **Desire** (sexual motivation): duration of partnership, imbalance of commitment, lifetime number of sexual partners. |  | **Desire** (sexual motivation): older age, living together, age at first coitus, church attendance, political orientation left to right, approval for feminist views. |
| Prevalence of male and female sexual problems, perceptions related to sex and association with quality of life in a Chinese population: A population-based study | Lau et al., 2005 [61] | **FSD**: older age, inadequacy of sex knowledge, belief that sex life is not very important or neutrality, low general life satisfaction, low mental health QOL score, low vitality QOL score, **Pain:**  low perceived health status, inadequate sexual knowledge, sexual dissatisfaction, belief that sexual life is not important or neutrality, low mental health QOL score **Lubrication**: 40-59 years old, married, div/wid/other, low perceived health status, inadequate sexual knowledge, low mental health QOL **Desire ("lack of interest"):** married, div/wid/other, belief that sex life is not very important or neutrality, low sexual satisfaction, low mental health QOL score, low general life satisfaction | **Pain**: married | **FSD**: educational level, full-time employment, marital status, chronic physical disease, perceived general health status, Lubrication: educational level **Orgasm:** older age, inadequate sexual knowledge, belief that sexual life is not important or neutrality, low vitality QOL **Desire ("lack of interest"):** older age, inadequacy of sex knowledge, perceived general health status, low vitality QOL |
| Prevalence and correlates of sexual dysfunction among young adult married women in rural China: A population-based study | Lau et al., 2006 [62] | **Pain:** education junior high school or above, average annual income 500-999 RMB Yuan, average annual income > 1000 RMB Yuan, sharing a bedroom with non-spouse family members; ever been pregnant, currently pregnant, age at menarche 13-14, age at menarche >15, whether using IUD, self-reporting menstruation disorder, ever having cervical erosion, experienced RTI, frequency of intercourse per week >2x per week masturbated in the last 12 months, belief that sex life is important **Orgasm:** education junior high school or above, average annual income 500-999 RMB Yuan, average annual income > 1000 RMB Yuan, sharing a bedroom with non-spouse family members, smoking, alcohol use, age at menarche 13-14, age at menarche >15, self-reporting menstruation disorder, ever having cervical erosion, experienced RTI, frequency of intercourse per week >2x per week masturbated in the last 12 months, belief that sex life is important **Desire**: education junior high school or above, age at first marriage > 25, average annual income 500-999 RMB Yuan, average annual income > 1000 RMB Yuan, sharing a bedroom with non-spouse family members, ever been pregnant, currently pregnant, age at menarche 13-14, age at menarche >15, self-reporting menstruation disorder, ever having cervical erosion, experienced RTI, frequency of intercourse per week >2x per week masturbated in the last 12 months, belief that sex life is important **Lubrication**: age at first marriage 20-24, average annual income 500-999 RMB Yuan, average annual income > 1000 RMB Yuan, sharing a bedroom with non-spouse family members, age at menarche 13-14, age at menarche >15, self-reporting menstruation disorder, ever having cervical erosion, experienced RTI, frequency of intercourse per week >2x per week masturbated in the last 12 months, belief that sex life is important |  | **Pain:** age at first marriage, smoking, alcohol use. **Orgasm:** age at first marraiage. |
| Hypoactive sexual desire disorder in postmenopausal women: US results from the Women's International Study of Health and Sexuality (WISHeS) | Leiblum et al., 2006[63] |  | **Desire**: higher frequency of intercourse, spontaneous sexual initiation, masturbation. |  |
| Risk factors for low sexual function among urban Chinese women: A hospital-based investigation | Lianjun et al., 2011 [64] | **FSD**: older age, low education, difficult delivery, depression, alcohol consumption, chronic disease, poor partner health, partner has SD, menopause, living separately from partner. | **FSD:** frequent communication with partner. | **FSD:** marital status, employment, having children, smoking, physical activity, attitude towards sex (positive / neg). |
| Sexual behavior and symptoms among reproductive age Chinese women in Hong Kong | Lo and Kok, 2014 [65] | **Desire**: low foreplay enjoyment, unidirectional coitus initiation, sexual intercourse less than 1x per month. **Arousal**: low foreplay enjoyment, high acceptance for pornography, neutral towards pornography, unidirectional coitus initiation, sexual intercourse less than 1x per month, chronic illness. **Orgasm**: low foreplay enjoyment, unidirectional coitus initiation, sexual intercourse less than 1x per month, primary education. **Pain**: unidirectional coitus initiation, sexual intercourse less than 1x per month, secondary education or less, planning to have more children. |  | **Desire**: level of education, attitudes towards pornography, chronic illness, planning to have more children. **Arousal**: level of education, planning to have more children. **Orgasm**: attitudes towards pornography, chronic illness, planning to have more children. **Pain**: foreplay enjoyment, attitudes towards pornography, chonic illness. |
| Prevalence and correlates of sexual activity and function in women: Results from the Boston Area Community Health (BACH) survey | Lutfey et al., 2009 [66] | **FSD:** race "white", older age, being married, sexual abuse, poor mental health. |  | **FSD:** SES, BMI, physical activity, alcohol consumption. |
| Prevalence of low sexual desire among women in Britain: Associated factors | Mitchell et al. 2009 [68] | **Desire**: older age, having children under 5, giving birth in past year. | **Desire**: single/never married. | **Desire**: ethnicity, self-perceived health status. |
| Sexual function in Britain: Findings from the third National Survey of Sexual Attitudes and Lifestyles (Natsal-3) | Mitchell et al. 2013 [67] | **FSD**: older age, unemployment, current depression, self-reported poor physical health, menopausal, no stead relationship -previously cohabitating, no steady relationship - never cohabitated, difficulty talking about sex with partners, relationship dissatisfaction, non-competence at first intercourse, not having four or more sexual acts in the previous 4 weeks, masturbating in the previous 4 weeks, no genital contact without intercourse in the previous 4 weeks, having at least one same sex partner in the previous 5 years, having 3-4, 5-9 or more than 10 partners during lifetime, ever having non-volitional sex, being diagnosed with an STI in the previous 5 years | **FSD:** pregnancy in the last year, steady relationship - not cohabitating |  |
| Prevalence and related factors for anorgasmia among reproductive aged women in Hesarak, Iran | Najafabady et al., 2011 [69] | **Orgasm**: older age, younger age at time of marriage, longer duration of marriage, less sex education during puberty, sexual dissatisfaction, belief that sex is a "duty", feelings of anxiety, fatigue, pain, guilt, anti-masculinity and embarrassment, absence of sexual pleasure. | **Orgasm**: higher education. | **Orgasm**: frequency of intercourse per month. |
| Use of the Italian translation of the Female Sexual Function Index (FSFI) in routine gynecological practice | Nappi et al., 2008 [70] | **FSD, Desire, Arousal, Lubrication, Orgasm, Pain** were significantly negatively associated with age. **FSD**: multiparity. **FSD**: menopause. | **FSD:** HRT. | **FSD:** using oral contraceptives. |
| Problems with sexual function in people attending London general practitioners: cross-sectional study | Nazareth et al., 2003 [71] | **FSD**: older age, unemployment, poor physical health, poor mental health, dissatisfaction with sex life. |  |  |
| Sexual activity and background variables among Finnish middle-aged women | Ojanlatva et al., 2004 [72] | **Desire**: being married / marriage-like relationship, having a spouse. |  | **Desire**: level of education, vocational education, residency (rural or urban). |
| Sexual problems among married Nigerian women | Ojomu et al., 2007 [73] | **Desire:** poor communication, chronic medical condition. **Arousal**: older age (46-55), polygamous marriage, chronic medical condition, current medication. **Pain**: gynecological condition. **Orgasm:** Islamic religion, polygamous marriage, occupation as housewife, forced sex by husband, poor communication. | **Desire**: some education. **Arousal**: some education. |  |
| Prevalence and risk factors for female sexual dysfunction in Turkish women | Oksuz and Malhan, 2006 [74] | **FSD:** older age, smoking, marital status, menopause, . |  | **FSD:** education, contraceptive use, chronic illness. |
| The prevalence and causes of sexual problems among premenopausal Turkish women | Oniz et al., 2007 [75] | **Pain**: low education | **Orgasm**: using contraceptives |  |
| Dyspareunia and urinary sensory symptoms in India: Population-based study | Padmadas et al., 2006 [76] | **Pain**: urinary sensory symptoms, nulliparity. |  | **Pain**: age at first union, duration of marriage. |
| Sexual dysfunctions in urban China: A population-based national survey of men and women | Parish et al., 2007 [77] | **Desire**: absence of daily intimacy, higher education, mental distress, fear of pregnancy, older age. **Pain**: varied sexual practices, finding sex "dirty", mental distress, poor health, older age. **Lubrication**: physical assault, unattractive partner, adulterous partner, finding sex "dirty", knowledge of clitoris, mental distress, older age. **Arousal**: absence of daily intimacy, physical assault, finding sex "dirty", higher education, liberal sex values, fear of pregnancy, genito-urinary symptoms, younger age. **Orgasm**: absence of daily intimacy, unattractive partner, adulterous partner, higher education, knowledge of clitoris, fear of pregnancy, job insecurity. |  | **Desire**: physical assault, knowledge of sexual needs, varied sexual practices, partner’s attractiveness, fidelity, sexual values, job security, overall health. **Pain**: physical assault, knowledge of sexual needs, partners attractiveness, fidelity, sexual values, job security. **Lubrication**: knowledge of sexual needs, varied sexual practices, level of education, overall health. **Arousal**: knowledge of sexual needs, varied sexual practices, partner’s attractiveness, fidelity, mental distress, job security. **Orgasm**: knowledge of sexual needs, sexual practices, sexual values, mental distress, overall health, age |
| Prevalence and socio-demographic predictors of sexual problems in Portugal | Peixoto and Nobre 2013 [78] | **FSD:** older age, low education. **Desire**: older age. **Orgasm**: older age. | **FSD:** level of education, marital status, duration of relationship. **Desire**: level of education, marital status, duration of relationship. **Arousal:** age, education, marital status, duration of relp. **Lubrication:** age, education, marital status. **Orgasm**: education, marital status, duration of relationship. **Pain:** age, education, marital status, duration of relationship. | **FSD:** marital status, duration of relationship. |
| Prevalence of sexual problems and associated distress among lesbian and heterosexual women | Peixoto and Nobre 2014 [79] |  | **Orgasm:** homosexual preference (lesbian). **Arousal**: lesbian. **Pain:** lesbian. | **Desire**: sexual preference (homo or hetero). |
| Sexual dysfunction, depression, and anxiety in young women according to relationship status: An online survey | Pereira et al., 2013 [80] | **FSD:** marital status (single), **Lubrication**: marital status (single), **Orgasm:** marital status (single), **Pain:** marital status (single). |  | **Desire**: marital status. **Arousal:** marital status. |
| Female sexual dysfunction in a healthy Austrian cohort: Prevalence and risk factors | Ponholzer et al., 2004 [81] | **Desire**: older age, low physical activity. **Arousal**: older age, low physical activity. **Orgasm**: stress. |  | **Desire**: BMI, education, alcohol, stress, smoking, hypertension, heart disease, diabetes, menopause, HRT. **Arousal**: BMI, education, alcohol, stress, smoking, hypertension, heart disease, diabetes, menopause, HRT. **Orgasm**: age, physical activity, BMI, education, alcohol, smoking, hypertension, heart disease, diabetes, menopause, HRT. |
| Pain at the vulvar vestibule: A web-based survey | Reed et al., 2004 82] | **Pain:** race |  |  |
| Sex in Australia: Sexual difficulties in a representative sample of adults | Richters et al., 2003 [83] | **FSD**: age 20-29, age 30-39, physical health reported as being bad, fair or good--versus very good or excellent, physical disability in the previous year. **Orgasm**: aged 50-59. **Lubrication:** older age. **Pain:** younger age. | **Desire:** aged -16-19. **Pain:** older age. | **FSD:** nationality, sexual identity, education, region of residence, household income, occupational classification, relationship status, medication for cardiovascular disease or hypertension, hyperglycemia/diabetes, regular sexual partner. |
| Female sexual dysfunction in a population-based study in Iran: Prevalence and associated risk factors | Safarinejad, 2006 [84] | **Desire:** older age, secondary education or less, unemployment, menopausal, having 3 or more children, having a chronic disease, marrying young (<= 18), having psychological problems, exercising 1x per week or less. **Arousal**: older age, primary school or less, menopausal, having a chronic disease, marrying young (<= 18), having psychological problems, exercising 1x per week or less. **Lubrication**: older age, secondary education or less, unemployment, menopausal, having a chronic disease, marrying young (<= 18), having psychological problems. **Orgasm**: older age, secondary education or less, unemployment, menopausal, having 3 or more children, having a chronic disease, marrying young (<= 18), having psychological problems, exercising 1x per week or less. **Pain**: primary education or less, menopausal, having a chronic disease, marrying young (<= 18), having psychological problems. | significant association between FSD and good physical health, between FSD and good mental health | **Desire**: contraceptive use, smoking history, previous pelvic surgery. **Arousal**: employment status, contraception use, number of children, smoking history, previous pelvic surgery. **Lubrication**: contraceptive use, number of children, smoking history, previous pelvic surgery, physical activity. **Orgasm**: contraceptive use, smoking history, previous pelvic surgery. **Pain**: employment status, contraception use, number of children, smoking history, previous pelvic surgery, physical activity. |
| Predicting sexual problems in women: The relevance of sexual excitation and sexual inhibition | Sanders et al., 2008 [85] | **Arousal:** full time employment, in a relationship. **Orgasm**: never reached orgasm, unsure if orgasm reached. **Desire**: full time employment, living with children, being married. | **Desire:** non-exclusive relationship. | **Arousal:** age. **Orgasm:** age. **Desire**: age. |
| The Global Online Sexuality Survey (GOSS): Female sexual dysfunction among Internet users in the reproductive age group in the Middle East | Shaeer et al., 2012 [86] | **FSD:** depression, low education, insufficient foreplay, masturbation, partner has ED, dissatisfaction with partner's penis size. |  | **FSD:** age, diabetes, hypertension, smoking pregnancy, mode of delivery, infertility, menstrual irregularities, dysmenorrhea, interpersonal distress, hirsutism (excessive hairiness), female genital mutilation. |
| Sexual problems and distress in United States women | Shifren et al., 2008 [87] | **Desire:** middle age group, being married, no current partner, employment, menopause, poor health, anxiety, thyroid problem, urinary incontinence, depression. **Arousal**: older age, being married, low education, menopause, poor health, arthritis, anxiety, thyroid problem, irritable bowel, urinary incontinence, depression. **Orgasm**: older age, low education, menopause, poor health, arthritis, anxiety, thyroid problem, urinary incontinence, depression. | **Desire**: African American race. **Orgasm:** African American race, being divorced. | **Desire:** education, HRT. **Arousal:** race, having a partner, employment, HRT. **Orgasm:** having current partner, employment, HRT. |
| An Internet survey of demographic and health factors associated with risk of sexual dysfunction in women who have sex with women | Shindel et al., 2012 [88] | **FSD:** older age, heart disease, depression, yeast infection, gynecological surgery, menopause, urinary incontinence, bisexual preference, having no partner, currently being with male partner. | **FSD:** Asian/Pacific Islander, higher frequency of sex (more than 3x / month). |  |
| The prevalence of sexual dysfunction and potential risk factors which may impair sexual function in Malaysian women | Sidi et al., 2007 [89] | **FSD:** Malaysian race, long duration of marriage, multiparity, older husband, higher education. | **FSD:** older age, high frequency of intercourse. | **FSD:** income, illness, contraceptives, HRT, smoking, alcohol. |
| Prevalence and risk factors for female sexual dysfunction in women attending a medical clinic in south India | Singh et al., 2009 [90] | **FSD:** older age, low education. |  | **FSD:** income, duration of marriage. |
| Rate and related factors of dyspareunia in reproductive age women: A cross-sectional study | Sobhgol et al., 2007 [91] | **Pain:** vaginal delivery, parity, cesarean, delivery without episiotomy, heavy lifting, chronic lung disease, pelvic organ prolapse, pelvic muscle strength, frequency of urinary infection >2x per year, stress incontinence, urgency, low back pain, history of pelvic inflammation disease, constipation, arthritis, low education. |  | **Pain:** age, delivery with episiotomy, BMI, weight of largest infant, genital hiatus, perineal diameter, depth of posterior fornix, exercise, employment status, hypertension, varicose, connective tissue disorder, familial history of pelvic floor dysfunction, previous pelvic surgery (hysterectomy, urinary incontinence surgery, pelvic organ prolapse surgery), uterus position, hemorrhoid, varicose, connective tissue disorder, familial history of pelvic floor dysfunction, pelvic organ prolapse |
| The prevalence and risk factors of female sexual dysfunction in young Korean women: An Internet-based survey | Song et al., 2008 [92] | **FSD:** older age, depression, negative attitude toward sex, history of sexual harassment, homosexuality, urinary problems (high IPSS score). | **FSD:** higher frequency of sex, masturbation. | **FSD:** IUD usage. |
| Dyspareunia, urinary sensory symptoms, and incontinence among young Chinese women | Stones et al., 2006 [93] | **Pain:** early sexual debut (<20), low education, not Han Chinese. |  | **Pain**: age, child birth / mode of delivery, rural or urban, occupation. |
| Sexual problems of urban women in Croatia: Prevalence and correlates in a community sample | Stulhofer et al., 2005 [94] | **Desire:** older age, length of relationship, religious morality. **Arousal:** age, religious morality. **Pain:** religious morality. | **Desire:** intimate communication. **Arousal:** intimate communication, positive body image. **Orgasm:** age, intimate communication. **Pain**: age, positive body image. | **Desire**: having children, body image. **Arousal:** length of relationship, having children. **Orgasm:** length of relationship, having children, religious morality, body image. **Pain**: length of relationship, having children, intimate communication. |
| Sexual health difficulties in a population-based sample of Croatian women aged 18-35 and the effects of the dual (career and motherhood) role | Stulhofer et al., 2011 [95] | **Desire:** older age, secondary education or less. **Lubrication:** secondary education or less. | **Desire:** intimate communication. **Lubrication:** intimate communication. **Orgasm**: being married/in a relationship, intimate communication. **Pain:** being married/in a relationship. | **Desire:** income, being married/in a relationship, employment status, dual role. **Lubrication**: age, income, being married/in a relationship, employment status, dual role. **Orgasm**: age, level of education, income, employment status, dual role. **Pain:** age, level of education, income, intimate communication, employment status, dual role. |
| Gynecologic pain related to occupational stress among female factory workers in Tianjin, China | Sznajder et al., 2014 [96] | **Pain**: irregular periods, worked overtime in the last 12 months, feeling exhausted. | **Pain:** excellent or good physical health. | **Pain**: age, marital status, having children, level of education, income, alcohol consumption, use of hormonal contraceptives, compulsory overtime, high noise level, high job strain, job type, heavy/light period, working late shifts, number of sick days taken. |
| Correlates of lesbian sexual functioning | Tracy and Junginger, 2007 [97] | **FSD**: psychological symptoms, dissatisfaction with relationship, duration of relationship (cut off not specified but negative correlation) |  |  |
| Reduced sexual desire in a random sample of Norwegian couples | Traeen et al., 2007 [98] | **Desire:** significant negative association between number of children, age of youngest child, satisfaction with how the family does house work, frequency of orgasm during sex, frequency of simultaneous orgasm during sex, significant positive association between habitualized negative thinking about the partner, habitualized negative thinking about oneself, negative work to home interference. |  | **Desire:** age, satisfaction with work-home division. |
| Help-seeking behaviors for female sexual dysfunction: A cross sectional study from Iran | Vahdaninia et al., 2009 [99] | **FSD:** low education, unemployment. |  |  |
| Prevalence of sexual dysfunction and its associated factors women aged 40-65 years with 11 years or more of formal education: a population-based study | Valadares et al., 2008 [100] | **FSD:** older age. | **FSD**: subjective good health, in partnership. |  |
| Sexual health in the Netherlands: Main results of a population survey among Dutch adults | Vanwesenbeeck et al., 2010 [101] | significant association between FSD and sexual violence | significant association between FSD and good physical health, between FSD and good mental health | Associations which were non-significant were not given in article. |
| Sexual dysfunction among young married women in southern India | Varghese et al., 2012 [102] | **FSD:** contraceptive use | **FSD:** higher income. |  |
| Orgasmic dysfunction: Prevalence and risk factors from a cohort of young females in Mexico | Villeda Sandoval et al., 2014 [103] | **Desire**: being unsatisfied with thickness/size of partner's penis | **Desire:** older age (30-40 vs. 18-29). | **Desire:** marital status, level of education, homosexual experience, frequency of sexual intercourse. |
| Prevalence of sexual dysfunction and impact of contraception in female German medical students | Wallwiener et al., 2010 [104] | **FSD**: smoking. **Desire**: hormonal contraception. **Arousal:** hormonal contraception. |  | **FSD**: age, former pregnancy, wish for children, partnership status. |
| Female sexual arousal disorder with and without a distress criterion: Prevalence and correlates in a representative Czech sample | Weiss and Brody, 2009 [105] | **Orgasm**: older age. |  |  |
| Female sexual dysfunction, sexual distress and compatibility with partner | Witting et al., 2008 [106] | **Desire, Arousal** and **Lubrication** were significantly negatively associated with age. **Desire, Arousal, Lubrication** and **Orgasm** were significantly negatively associated with longer duration of relationship. | **Orgasm** and **Pain** had positive association with age. **Pain**: positive association with longer duration of relationship. |  |
| Sexual dysfunction among women of low-income status in an urban setting | Worly et al., 2010 [107] | **FSD:** older age, unemployment, African American race, sleeping problems, urinary incontinence, depression, polypharmacy. |  | **FSD:** relationship status, income, level of education, BMI, parity, diabetes, hysterectomy |
| Sexual dysfunction and related risk factors in a cohort of middle-aged Ecuadorian women | Yanez et al., 2006 [108] | **FSD:** partner with SD, antidepressant use, being married. | **FSD:** employment, high education, frequent intercourse (4x/month or more), only 1 sexual partner. | **FSD:** church attendance. |
| Female sexual dysfunction among young and middle-aged women in Hong Kong | Zhang and Yip, 2012 [109] | **Desire**: being married more than once, sex perceived as unimportant, dissatisfied in marriage. **Lubrication:** high education, infertility, seeking medical help, partner has SD. **Orgasm:** being married more than once, had an abortion, seeking medical help, partner has premature ejaculation, sex perceived as unimportant. **Pain:** high education, infertility, had an abortion, seeking medical help. | **Desire:** liberal attitudes towards sex. **Pain**: liberal attitudes towards sex. | **Desire**: level of education, having children, employment status, being religious, sexual experience factors. **Lubrication:** being married more than once, having children, employment status, being religious, had an abortion, partner has premature ejaculation, sex attitudes, satisfaction with marriage. **Orgasm:** level of education, being married more than once, having children, being religious, infertility, had an abortion, partner has SD, sex attitudes, satisfaction with marriage. |
