# Supplementary material for: Predictors of female sexual dysfunction: a systematic review and qualitative analysis through gender inequality paradigms
Source: BMC Womens Health. 2018 Jun 22;18:108. doi: 10.1186/s12905-018-0602-4 (PMC6013982; doi:10.1186/s12905-018-0602-4)
Supplement: Supplementary file 2 — Supplementary Material. Significant factors stratified by sexual regime. (DOCX 22 kb) [file 12905_2018_602_MOESM2_ESM.docx]

**GENDER EQUAL**

FSD RISK: smoking, relationship dissatisfaction, older age, unemployment, current depression, self-reported poor physical health, menopausal, no stead relationship - previously cohabitating, no steady relationship - never cohabitated, difficulty talking about sex with partners, relationship dissatisfaction, non-competence at first intercourse, not having four or more sexual acts in the previous 4 weeks, masturbating in the previous 4 weeks, no genital contact without intercourse in the previous 4 weeks, having at least one same sex partner in the previous 5 years, having 10 or more sexual partners during lifetime, rape, being diagnosed with an STI in the previous 5 years, older age, unemployment, poor physical health, poor mental health, sex life dissatisfaction, multiparity, urinary incontinence, menopause, anti-depressants, race “white” (in USA), being married, sexual abuse, poor mental health, younger age, reported poor health, physical disability in previous year, heart disease, depression, yeast infection, gynecological surgery, bisexual preference, no partner, psychological symptoms, long duration of relationship, unemployment, “African American” (in USA), sleeping problems, polypharmacy

FSD PROTECTIVE: emotional intelligence, pregnancy in last year, steady relationship – not cohabitating, “Asian/Pacific Islander” (in USA), higher frequency of sex intercourse (>3x/month)

**GENDER EQUAL**

DESIRE RISK: older age, low physical activity, lack of partnership, childhood sexual abuse, long duration of partnership, hormonal contraceptives, having children under 5, giving birth in past year, symptoms of depression, genito-urinary symptoms, unemployment, multiparity, menopause, “Chinese” (in USA), “Japanese” (in USA), never being married, economic hardship, “sex is not important”, dissatisfaction with partner, full-time employment, living with children, being married, employment, poor health, anxiety, thyroid problem, urinary incontinence, depression

DESIRE PROTECTIVE: imbalance of commitment (woman more committed than man), higher no. of lifetime sexual partners, masturbation, single / never married, smoking, alcohol consumption, exercising 2x / week, higher frequency of intercourse, spontaneous sexual initiation, masturbation, non-exclusive relationship, age 16-19, “African American” (in USA)

**GENDER EQUAL**

AROUSAL RISK: older age, low physical activity, hormonal contraceptive, “African American” “Hispanic” “Chinese” in the USA, menopause, depression, low education, “sex is not important”, full time employment, in a relationship, being married, low education, menopause, poor health, arthritis, anxiety, thyroid problems, irritable bowel, urinary incontinence, depression

AROUSAL PROTECTIVE: emotional intelligence, never married, widowed / divorced / separated, higher education, middle age (30-49), HRT

**GENDER EQUAL**

LUBRICATION RISK: older age, relationship dissatisfaction

LUBRICATION PROTECTIVE: -

**GENDER EQUAL**

ORGASM RISK: stress, older age, relationship dissatisfaction, “sex is not important”, never/unsure if reached orgasm, being unsatisfied with size/thickness of partner’s penis

ORGASM PROTECTIVE: middle age (30-39), age 30-40 vs. 18-29

**GENDER EQUAL**

PAIN RISK: early sexual debut (<15y), hormonal contraceptives, younger age, relationship dissatisfaction, anxiety, low ecucation, unemployment, in partnership, menopause, urinary incontinence, perimenopausal, “African American” “Chinese” in USA, never married, divorced /separated / widowed), older age, c-section, IUD, STI, colitis, chronic UTI, younger age

PAIN PROTECTIVE: regular / frequent sexual intercourse, current employment, older age

**MIXED MALE-CENTERED**

FSD RISK: single, older age, medical illness, menopause, low frequency of intercourse, Malaysian race (in Malaysia), long duration of marriage, multiparity, older husband, higher education, older age, low education, menopause, depression, partner has SD, older age, low education, unemployment, chronic illness, multiparity, menopause, young age at marriage, higher no. of births, higher no. of children, unemployment, rural living, low ed, low education of husband, parents with restrictive attitudes, genital infections, arranged marriage, older age, smoking, married, menopause, dieting, married more than 10 years, female genital mutilation, partner over 50, irregular periods, nulliparity, multiparity, negative attitude toward sex, sexual harassment, homosexuality, urinary problems

FSD PROTECTIVE: perceived good health, in partnership, older age, higher frequency of intercourse, using some form of contraceptive, sex education, HRT

**MIXED MALE-CENTERED**

DESIRE RISK: low frequency of intercourse, partner with SD, long duration of marriage, older than 26, low education, race “brown” (in Brazil), older age, widowed, low education, multiparity, unemployment, cardiovascular disease, diabetes, breast cancer, PTSD, hypertension, depression, low hormones, drug addiction, late sexual debut, only 1 significant lifetime sexual partner, no / too little foreplay, older age,

DESIRE PROTECTIVE: unmarried, single, divorced, moderate alcohol consumption, sex education, spontaneous sexual initiation, varied sexual repertoire

**MIXED MALE-CENTERED**

AROUSAL RISK: single, older age

AROUSAL PROTECTIVE: -

**MIXED MALE-CENTERED**

LUBRICATION RISK: single, older age

LUBRICATION PROTECTIVE: -

**MIXED MALE-CENTERED**

ORGASM RISK: single, older age, low education

ORGASM PROTECTIVE: using contraceptives, unmarried

**MIXED MALE-CENTERED**

PAIN RISK: single, low education, unmarried, low education, older age

PAIN PROTECTIVE: older age, race “brown” (in Brazil)

**ASIAN MALE CENTERED**

FSD RISK: older age, lack sex knowledge, “sex not important”/ neutrality, low life satisfaction, low mental health score, low vitality score, low education, difficult delivery, depression, alcohol consumption, chronic illness, poor partner health, partner has SD, menopause, living separate from partner, lower education, older age, mid to upper income, sharing a bedroom with non-spouse family members, smoking, alcohol, ever pregnant, late debut menarche, using IUD, menstruation disorder, cervical erosion, experienced RTI, masturbated in last 12 months.

FSD PROTECTIVE: frequent cx with partner, “sex is important”

**ASIAN MALE CENTERED**

DESIRE RISK: married, divorced/widowed, “sex is not important” / neutrality, low sexual satisfaction, low mental health, low life satisfaction, low foreplay enjoyment, unidirectional coital initiation, sexual inter less than 1x month, no daily intimacy, higher education, mental distress, fear of pregnancy, older age, being married more than once, sex “unimportant”, dissatisfied in marriage, parity, married 6-10 years (vs <5 or >10), diabetes, poor relationship with partner, no steady partner, partner has ED, education junior high school or above, age at first marriage > 25, average annual income 500-999 RMB Yuan, average annual income > 1000 RMB Yuan, sharing a bedroom with non-spouse family members, ever been pregnant, age at menarche 13-14, age at menarche >15 (late debut menarche), self-reporting menstruation disorder, ever having cervical erosion, experienced RTI, frequency of intercourse per week >2x per week, masturbated in the last 12 months, older age

DESIRE PROTECTIVE: liberal attitudes toward sex, (older age at marriage), currently pregnant, belief that sex life is important

**ASIAN MALE CENTERED**

AROUSAL RISK: low foreplay enjoyment, high acceptance for porn, neutral towards porn, unidirectional coital initiation, sex inter less 1x/month, chronic illness, no daily affection, physical assault, finding sex “dirty”, higher education, liberal sex values, fear of pregnancy, genito-urinary problems, younger age, older age, older age, poor relationship with partner, partner has SD

AROUSAL PROTECTIVE: -

**ASIAN MALE CENTERED**

LUBRICATION RISK: older age, married, divorced/widowed, low perceived health, lack sex knowledge, low mental health, physical assault, unattractive partner, adulterous partner, finding sex “dirty”, knowledge of clitoris, mental distress, older age, high education, infertility, seeking medical help, partner has SD, older age, no steady partner, partner has SD, urinary incontinence, education junior high school or above, age at first marriage over 20, average annual income 500-999 RMB Yuan, average annual income > 1000 RMB Yuan, sharing a bedroom with non-spouse family members, age at menarche 13-14, age at menarche >15 (late debut menarche), self-reporting menstruation disorder, ever having cervical erosion, experienced RTI, frequency of intercourse per week >2x per week, masturbated in the last 12 months.

LUBRICATION PROTECTIVE: older age at marriage, belief that sex life is important.

**ASIAN MALE CENTERED**

ORGASM RISK: low foreplay enjoyment, unidirectional coital initiation, sex inter less 1x/month, low education, no daily affection, unattractive partner, adulterous partner, higher education, knowledge of clitoris, fear of pregnancy, job insecurity, being married more than once, abortion, seeking medical help, partner has SD, “sex is unimportant”, poor relp with partner, partner has SD, education junior high school or above, average annual income 500-999 RMB Yuan, average annual income > 1000 RMB Yuan, sharing a bedroom with non-spouse family members, smoking, alcohol use, age at menarche 13-14, age at menarche >15 (late debut menarche), self-reporting menstruation disorder, ever having cervical erosion, experienced RTI, frequency of intercourse per week >2x per week, masturbated in the last 12 months, older age

ORGASM PROTECTIVE: married 5 years or less, “sex is important”

**ASIAN MALE CENTERED**

PAIN RISK: low perceived health status, lack sex knowledge, sexual dissatisfaction, “sex not important” / neutrality, low mental health score, unidirectional coital initiation, sex inter less 1x/month, low education, planning to have more children, varied sexual practices, finding sex “dirty”, mental distress, poor health, older age, early sexual debut, low education, not Han Chinese (living in China), irregular periods, worked overtime, exhaustion, high education, infertility, abortion, seeking medical help, younger age, urinary incontinence, no steady partner, education junior high school or above, average annual income 500-999 RMB Yuan, average annual income > 1000 RMB Yuan, sharing a bedroom with non-spouse family members; ever been pregnant, age at menarche 13-14, age at menarche >15 (late debut menarche), using IUD, self-reporting menstruation disorder, ever having cervical erosion, experienced RTI, frequency of intercourse per week >2x per week masturbated in the last 12 months, older age

PAIN PROTECTIVE: married, good physical health, liberal attitudes towards sex, currently pregnant, belief that sex life is important
