# Supplementary material for: Predictors of female sexual dysfunction: a systematic review and qualitative analysis through gender inequality paradigms
Source: BMC Womens Health. 2018 Jun 22;18:108. doi: 10.1186/s12905-018-0602-4 (PMC6013982; doi:10.1186/s12905-018-0602-4)
Supplement: Supplementary file 3 — Supplementary Material. Significant risk factors stratified by level of human development. (DOCX 27 kb) [file 12905_2018_602_MOESM3_ESM.docx]

**VERY HIGH HUMAN DEVELOPMENT**

**FSD:** age 20-29, age 30-39, physical health reported as being bad, fair or good--versus very good or excellent, physical disability in the previous year, smoking, relationship dissatisfaction, older age, unemployment, current depression, self-reported poor physical health, menopausal, no stead relationship -previously cohabitating, no steady relationship - never cohabitated, difficulty talking about sex with partners, relationship dissatisfaction, non-competence at first intercourse, not having four or more sexual acts in the previous 4 weeks, masturbating in the previous 4 weeks, no genital contact without intercourse in the previous 4 weeks, having at least one same sex partner in the previous 5 years, having 10 or more sexual partners during lifetime, rape, being diagnosed with an STI in the previous 5 years , older age, unemployment, poor physical health, poor mental health, dissatisfaction with sex life, multiparity, urinary incontinence, age, menopause, SSRI usage (selective serotonin reuptake inhibitors), race "white", older age, being married, sexual abuse, poor mental health, older age, heart disease, depression, yeast infection, gynecological surgery, menopause, urinary incontinence, bisexual preference, having no partner, currently being with male partner, psychological symptoms, dissatisfaction with relationship, duration of relationship (cut off not specified but negative correlation), partner has ED, hysterectomy, menopause, age > 49 years, older age, unemployment, African American race, sleeping problems, urinary incontinence, depression, polypharmacy, older age, depression, negative attitude toward sex, history of sexual harassment, homosexuality, urinary problems (high IPSS score), older age, multiparity, menopause, living in city with more than 100,000 inhabitants (urban living), more than 12 years of school attendance, high further education, economic hardship in the family, unmarried, older age, older age, low education

**Desire:** older age, low physical activity, older age, lack of partnership, childhood sexual abuse, (sexual motivation): longer duration of partnership, hormonal contraception, older age, having children under 5, giving birth in past year, having symptoms of depression, genitourinary symptoms, older age, unemployment, having children, menopausal, ethnicity (Chinese, Japanese), older age, never being married, paying for basics is hard, paying for basics is moderately hard, unemployment, sex is not important, in relationship for 20-29 years, dissatisfaction with partner, full time employment, living with children, being married, middle age group, being married, no current partner, employment, menopause, poor health, anxiety, thyroid problem, urinary incontinence, depression, older age, older age, older age, length of relationship, religious morality, older age, low SES, sexual abuse, anxiety, emotional problems, being married / marriage-like relationship, having a spouse, having young children, habitualized negative thinking about the partner, habitualized negative thinking about oneself, dissatisfaction about how housework is done, older age, older age. Desire: internal stress (within couple), older age, secondary education or less, older age, long duration of relationship.

**Lubrication and Arousal**: older age. Arousal: older age, low physical activity. Arousal: hormonal contraception. Arousal: older age. Lubrication: older age, relationship dissatisfaction. Arousal: race (African American, Hispanic, Chinese). Arousal: perimenopausal, postmenopausal, depression, low education, sex is not important. Arousal: full time employment, in a relationship. Arousal: older age, being married, low education, menopause, poor health, arthritis, anxiety, thyroid problem, irritable bowel, urinary incontinence, depression. Arousal: older age. Lubrication: older age. Arousal: older age. Lubrication: older age. Lubrication: secondary education or less. Arousal: age, religious morality. Lubrication: non or semi-skilled worker, difficulties paying bills during the previous year. Arousal and Lubrication were significantly negatively associated with age. Arousal and Lubrication were significantly negatively associated with longer duration of relationship. Arousal: older age. Lubrication: older age. Arousal: poor relationship quality, internal stress.

**Orgasm:** aged 50-59, stress, older age, relationship dissatisfaction, sex is not important, never reached orgasm, unsure if orgasm reached, older age, low education, menopause, poor health, arthritis, anxiety, thyroid problem, urinary incontinence, depression, older age, older age, older age, living in a city with more than 100,000 inhabitants (urban living), unmarried women, was significantly negatively associated with longer duration of relationship, older age, critical life event.

**Pain:** younger age, regular sexual intercourse before the age of 15 years or earlier, using oral contraceptives for more than 2 years, younger age (20-29, 30-39), relationship dissatisfaction, anxiety, low educational attainment, working status, with a partner, menopausal/HRT, urinary incontinence, perimenopause, race (African American, Chinese) marital status (never married, widowed/separated/divorced), older age, older age, relgious morality, living in a city with more than 100,000 inhabitants (urban living).

**HIGH HUMAN DEVELOPMENT**

**FSD:** older age, inadequacy of sex knowledge, belief that sex life is not very important or neutrality, low general life satisfaction, low mental health QOL score, low vitality QOL score, older age, low education, difficult delivery, depression, alcohol consumption, chronic disease, poor partner health, partner has SD, menopause, living separately from partner, lower education, marital status (single), older age, having a medical illness, menopause, low frequency of intercourse. Malaysian race, long duration of marriage, multiparity, older husband, higher education, older age, low education (less than 8 yrs), menopause, depression, partner has SD, older age, low education, unemployment, chronic disease, multiparity, menopause, younger age at marriage, higher number of births, higher number of children, housewife (unemployment), rural residence, low education, low education level of the husband, being brought up by parents with restrictive attitudes, having genital infections or symptoms, arranged marriage, older age, smoking, marital status, menopause, average annual income 500-999 RMB Yuan, average annual income > 1000 RMB Yuan, sharing a bedroom with non-spouse family members, smoking, alcohol, ever pregnant, late debut menarche, using IUD, menstruation disorder, cervical erosion, experienced RTI, masturbated in last 12 months, elementary education, high school education, not living together, depressive feelings, current use of antidepressives, partner with SD, antidepressant use, being married, older age, low education, low education of husband, unemployment, unemployment of husband, low SES, smoking, husband smokes, older age, lower education, sexual intercourse less than 3x per week, having 3 or more children, having a husband age 40 or more, being married 10 years or more, unemployment, low education, unemployment, older age, obesity, higher education.

**Desire:** married, divorced/widowed/other, belief that sex life is not very important or neutrality, low sexual satisfaction, low mental health QOL score, low general life satisfaction, low foreplay enjoyment, unidirectional coitus initiation, sexual intercourse less than 1x per month, absence of daily intimacy, higher education, mental distress, fear of pregnancy, older age, being married more than once, sex perceived as unimportant, dissatisfied in marriage, parity, married 6-10 years (vs. <5 or >10), diabetes, poor relationship with partner, no steady partner, partner has ED, aged 26 to 40, aged 41 or older, only high school education, only elementary education, race "brown", older age, widowed, low education, having children, unemployment, cardiovascular disease, diabetes, breast cancer, PTSD, hypertension, depression, low hormone levels, excessive use of medication (addiction), first sexual intercourse after age 21, only one significant sexual partner in lifetime, no foreplay or too little foreplay, low frequency of intercourse, partner with ED, longer duration of marriage, education junior high school or above,age at first marriage > 25, average annual income 500-999 RMB Yuan, average annual income > 1000 RMB Yuan, sharing a bedroom with non-spouse family members, ever been pregnant, age at menarche 13-14, age at menarche >15 (late debut menarche), self-reporting menstruation disorder, ever having cervical erosion, experienced RTI, frequency of intercourse per week >2x per week, masturbated in the last 12 months, urban, low education, low education of husband, unemployment of husband, low SES,older age, tubal ligation, older age, secondary education or less, unemployment, menopausal, having 3 or more children, having a chronic disease, marrying young (<= 18), having psychological problems, exercising 1x per week or less.

**Lubrication and Arousal:** 40-59 years old, married, divorced/widowed/other, low perceived health status, inadequate sexual knowledge, low mental health QOL. Arousal: low foreplay enjoyment, high acceptance for pornography, neutral towards pornography, unidirectional coitus initiation, sexual intercourse less than 1x per month, chronic illness. Arousal: absence of daily intimacy, physical assault, finding sex "dirty", higher education, liberal sex values, fear of pregnancy, genito-urinary symptoms, younger age. Lubrication: physical assault, unattractive partner, adulterous partner, finding sex "dirty", knowledge of clitoris, mental distress, older age. Lubrication: high education, infertility, seeking medical help, partner has SD. Arousal: older age, poor relationship with partner, partner has ED, partner has PE, partner has low desire. Lubrication: older age, urinary incontinence, no steady partner, partner has ED. Arousal: marital status (single). Lubrication: marital status (single), Lubrication: education junior high school or above, age at first marriage over 20, average annual income 500-999 RMB Yuan, average annual income > 1000 RMB Yuan, sharing a bedroom with non-spouse family members, age at menarche 13-14, age at menarche >15 (late debut menarche), self-reporting menstruation disorder, ever having cervical erosion, experienced RTI, frequency of intercourse per week >2x per week, masturbated in the last 12 months. Arousal: low education, low education of husband, unemployment, unemployment of husband, low SES. Lubrication: low education, low education of husband, unemployment, unemployment of husband, low SES. Arousal: older age. Arousal: older age, primary school or less, menopausal, having a chronic disease, marrying young (<= 18), having psychological problems, exercising 1x per week or less. Lubrication: older age, secondary education or less, unemployment, menopausal, having a chronic disease, marrying young (<= 18), having psychological problems.

**Orgasm:** low foreplay enjoyment, unidirectional coitus initiation, sexual intercourse less than 1x per month, primary education, absence of daily intimacy, unattractive partner, adulterous partner, higher education, knowledge of clitoris, fear of pregnancy, job insecurity, being married more than once, had an abortion, seeking medical help, partner has premature ejaculation, sex perceived as unimportant, poor relp with partner, partner has ED, partner has PE, being unsatisfied with thickness/size of partner's penis, aged 41 or older, unmarried, only high school education, only elementary education, marital status (single), Pain: marital status (single), education junior high school or above, average annual income 500-999 RMB Yuan, average annual income > 1000 RMB Yuan, sharing a bedroom with non-spouse family members, smoking, alcohol use, age at menarche 13-14, age at menarche >15 (late debut menarche), self-reporting menstruation disorder, ever having cervical erosion, experienced RTI, frequency of intercourse per week >2x per week, masturbated in the last 12 months, low education, low education of husband, unemployment, unemployment of husband, low SES, smoking, housewife or manual laborer, older age, younger age at time of marriage, longer duration of marriage, less sex education during puberty, sexual dissatisfaction, belief that sex is a "duty", feelings of anxiety, fatigue, pain, guilt, anti-masculinity and embarrassment, absence of sexual pleasure, older age, seconardy education or less, unemployment, menopausal, having 3 or more children, having a chronic disease, marrying young (<= 18), having psychological problems, exercising 1x per week or less. Pain: primary education or less, menopausal, having a chronic disease, marrying young (<= 18), having psychological problems.

**Pain:** low perceived health status, inadequate sexual knowledge, sexual dissatisfaction, belief that sexual life is not important or neutrality, low mental health QOL score, unidirectional coitus initiation, sexual intercourse less than 1x per month, secondary education or less, planning to have more children, varied sexual practices, finding sex "dirty", mental distress, poor health, older age, early sexual debut (<20), low education, not Han Chinese, irregular periods, worked overtime in the last 12 months, feeling exhausted, high education, infertility, had an abortion, seeking medical help, younger age, urinary incontinence, no steady partner, unmarried, only high school education, only elementary education, low education, education junior high school or above, average annual income 500-999 RMB Yuan, average annual income > 1000 RMB Yuan, sharing a bedroom with non-spouse family members; ever been pregnant, age at menarche 13-14, age at menarche >15 (late debut menarche), using IUD, self-reporting menstruation disorder, ever having cervical erosion, experienced RTI, frequency of intercourse per week >2x per week masturbated in the last 12 months, low education of husband, unemployment, unemployment of husband, low SES, husband smokes, nulliparity, having 1-3 deliveries, low education, vaginal delivery, multiparity, cesarian, delivery without episiotomy, heavy lifting, chronic lung disease, pelvic organ prolapse, pelvic muscle strength, frequency of urinary infection >2x per year, stress incontinence, urgency, low back pain, history of pelvic inflammation disease, constipation, arthritis, low education.

**MEDIUM AND LOW HUMAN DEVELOPMENT**

**FSD (medium HD):** female genital mutilation, older age, secondary education, female genital mutilation, abnormal menstrual pattern, nulliparity, multiparity, older age, multiparity, married more than 10 years, menopause, female genital mutilation. older age, menopausal, married over 10 years, female genital mutilation, partner over 50 years old, partner has SD, multiparity, mental stress, partner has SD. alcohol, non-sensuality, long duration of marriage (13+ yrs). illiteracy, lower education level, chronic disease, menopause, pelvic inflammatory disease, endometriosis, 24-30 years, 38-42 years, older than 42 years: older age, low education, contraceptive use.

**FSD (low HD):** tertiary education higher education), history of sexual abuse, guilt feelings about previous abortions, medications, current illness, polygamous relationship.

**Desire:** poor communication, chronic medical condition.

**Lubrication and Arousal:** Arousal: older age (46-55), polygamous marriage, chronic medical condition, current medication.

**Orgasm:** being single, Islamic religion, polygamous marriage, occupation as housewife, rape by husband, poor communication, non-sensuality.

**Pain:** being married, gynaecological condition, (vaginismus): increased with non-sensuality and infrequency and older age, urinary sensory symptoms, nulliparity.
